# Targets for intervention to prevent substance use in young people exposed to childhood adversity: A systematic review

**DOI:** 10.1371/journal.pone.0252815

**Published:** 2021-06-07

**Authors:** Lucinda Grummitt, Erin Kelly, Emma Barrett, Katherine Keyes, Nicola Newton

**Affiliations:** 1 NHMRC Centre of Research Excellence PREMISE, The Matilda Centre for Research in Mental Health and Substance Use, Sydney Medical School, The University of Sydney, Sydney, Australia; 2 Department of Epidemiology, Mailman School of Public Health, Columbia University, New York, New York, United States of America; Xiamen University - Malaysia Campus: Xiamen University - Malaysia, MALAYSIA

## Abstract

**Background and aims:**

Childhood adversity is a strong, and concerningly prevalent, risk factor for the later development of substance misuse. Yet despite substantial accumulating evidence for causal mechanisms, there has been little attempt to synthesize the strength of the evidence. Importantly, these mechanisms may be amenable to intervention, providing targets for substance use prevention among those exposed to childhood adversity. The present review aimed to systematically identify mediating and moderating mechanisms operating between childhood adversity and substance use.

**Methods:**

A systematic review was conducted. Electronic databases (PubMed, MEDLINE, PsycINFO, Web of Science and CINAHL) were searched from 1998 to 2020 for modifiable mediators and moderators of the relationship between childhood adversity and substance use in people aged 10–24. Data was qualitatively synthesised, using a socio-ecological perspective to group mediators/moderators into individual, interpersonal, community, and public policy/cultural levels of behaviour.

**Results:**

After screening against eligibility criteria, 50 studies were included in the current review. The mediators at the individual level of behaviour showing the largest and most consistent effect sizes included externalising behaviour, anger, coping motives for substance use, and post-traumatic stress symptoms. Among individual-level moderators, religiosity, future orientation and depressive symptoms all attenuated the relationship between childhood adversity and substance use. At the interpersonal level, peer relationships and mother-child relationships mediated the effect of adversity on substance use. Moderators included family cohesion and relationship quality. Community factors were less commonly studied, though school mobility and educational achievement mediated 14% and 28% of the total effect of childhood adversity on substance use respectively. No mediators or moderators were identified for public policy/culture.

**Conclusions:**

A substantial proportion of the relationship between childhood adversity and substance use in youth is mediated through individual, interpersonal and community factors. Coupled with the knowledge that existing, evidence-based programs effectively address many of the identified mediators and moderators, this review advances knowledge on optimal targets to prevent substance misuse among those exposed to childhood adversity.

## Introduction

Over one quarter of all cases of substance use disorder can be attributed to experiencing childhood adversity [1]. This reflects a substantially increased risk of harmful substance use [2,3] and substance use disorder [4] for children exposed to childhood adversity, compared to their non-exposed peers. Despite some variation in definitions, childhood adversity is viewed as encompassing significant threat or deprivation (see [5]), stemming from ten adverse childhood experiences (ACEs) that include physical, sexual, or emotional abuse, physical or emotional neglect, parent mental illness, household substance use, household incarceration or household violence [6]. Prevalence estimates suggest over one third of children have experienced an ACE; approximately two in five of those are exposed to multiple types [1]. For children exposed to four or more different types of ACEs, the odds for problematic drinking are six times higher and ten times higher for problematic drug use than those with no ACE exposure [7]. This highlights a substantial opportunity to intervene to prevent the large individual and social burden associated with substance use disorders [8,9]. Although preventing ACEs is an ultimate goal, given that it is not always possible, efforts to prevent the negative consequences of exposure such as substance misuse are of vital importance.

Effective prevention of substance use problems must occur early, prior to the development of harmful, chronic patterns of use. In this respect, adolescence represents a critical period. During this formative period spanning from approximately age 10–24 years [10], substance use typically begins and escalates [11,12], and approximately three quarters of lifetime cases of substance use disorder have their onset prior to age 24 [13]. Thus, examining mechanisms linking ACEs and substance use has the greatest relevance for prevention if outcomes are measured by early adulthood.

The socio-ecological perspective provides a useful framework for considering the factors involved in preventing harmful substance use [14]. Rather than focusing on solely the individual as responsible for health-harming behaviours, it includes social and environmental factors as targets for intervention. Specifically, it identifies influences at the individual, interpersonal, community, and public policy/culture levels of behaviour. These levels can be used to conceptualise the mechanisms that link ACEs and substance use, representing possible targets for intervention to prevent harmful substance use. These targets could be identified as factors that mediate or moderate the association between ACEs and substance use. For example, at the interpersonal level, evidence shows children exposed to ACEs receive less parental monitoring and have a less-supportive relationship with parents, in turn leading to substance use in adolescence [15].

To date, no synthesis of these mediating/moderating factors has been undertaken to weight the strength of the evidence. Existing studies have typically examined a single mediator or moderator of this relationship, or a group at one level of the socio-ecological model. While of value, identifying a single mediator or moderator may be missing multiple mechanisms that contribute to the relationship and could be targeted for prevention. This is important, as variations in the type of exposure, the child’s response to exposure, and their contact with intervention services or protective factors, can vary greatly, impacting subsequent development. Thus, synthesising available evidence on the potentially broad range of mediating and moderating factors maximises the potential to develop effective prevention strategies for children with varying experiences and contexts. Moreover, while effective substance use prevention exists, often in the form of school-based programs [16,17], it is unknown whether these programs are similarly effective for young people exposed to ACEs. An understanding of mediators and moderators could inform necessary adaptations to existing substance use prevention programs and development of new trauma-informed programs. Thus, through a systematic review of the literature, the current study aims to identify and synthesise the modifiable factors that mediate or moderate the relationship between ACEs and substance use in young people.

## Method

This protocol adheres to the Preferred Reporting Items for Systematic Review and Meta-Analysis Protocols (PRISMA-P) [18] and a checklist is provided in the Supporting Information. The protocol has been published elsewhere [19] was pre-registered in the PROSPERO registry (University of York, registration: CRD42020148773, https://www.crd.york.ac.uk/prospero/display_record.php?RecordID=148773).

### Search strategy and eligibility

Electronic searches were conducted in PubMed, MEDLINE, PsycINFO, Web of Science and CINAHL from 1 January 1998 to 14 August 2019. Searches were repeated on 11 June 2020 to capture any articles published since the initial searches were carried out. Two relevant journals were hand searched from 1 January 2011 to 8 June 2020 to promote retrieval of studies not identified by electronic searches.

Search terms are provided in Supporting Information. Databases were searched for studies conducted with human participants exposed to ACEs between age 0 and 18. ACEs were defined as emotional, physical or sexual abuse, emotional or physical neglect, mother treated violently, a member of the household engaged in substance abuse, experienced mental illness, or went to prison, parents were separated or divorced [6], being a victim of bullying, experiencing social isolation/rejection or prolonged loneliness [20]. For the current review, parental psychopathology must have occurred during the child’s lifetime between age 0–18.

Only studies that had an outcome measure of substance use between age 10–24 and included a mediation/moderation analysis of at least one factor that is modifiable through psychosocial intervention were eligible. Studies were required to report a test of the indirect effect from an ACE to the substance use outcome via a hypothesised mediator. For moderation, studies were required to test the interaction between an ACE and the proposed moderator. Peer reviewed, longitudinal studies reporting original research were included. Full details of the search strategy and inclusion criteria are available in the PROSPERO protocol https://www.crd.york.ac.uk/prospero/display_record.php?RecordID=148773.

Reviewer one (LG) screened 100% of titles and abstracts for inclusion in the review. Reviewers two (EK) and three (NN) each screened 5% of the titles and abstracts to ensure accuracy in study inclusion. Reviewer one (LG) assessed all full-text studies for inclusion. Reviewer two (EK) assessed 40%, reviewer three (NN) assessed 24% and reviewer four (EB) screened 36% of articles, ensuring every full-text article was evaluated by two reviewers. Inter-rater reliability was moderate (agreement ranging from 79% to 84% between reviewers), Cohen’s kappa = 0.36–0.55. Discrepancies were resolved through consultation between the reviewers.

### Data synthesis

Study information, substance use outcomes, ACE exposure, mediators/moderators and effect estimates for the indirect (mediated) effect and interaction (moderated) effect were extracted by reviewer one. Results of mediation/moderation analyses were classified by level of the socio-ecological model [14]. A qualitative synthesis was conducted. The data precluded meta-analysis due to the effect size coefficient (standardised betas controlling for different covariates) and an insufficient number of studies examining the same mediator and child adversity exposure.

**Fig 1:**
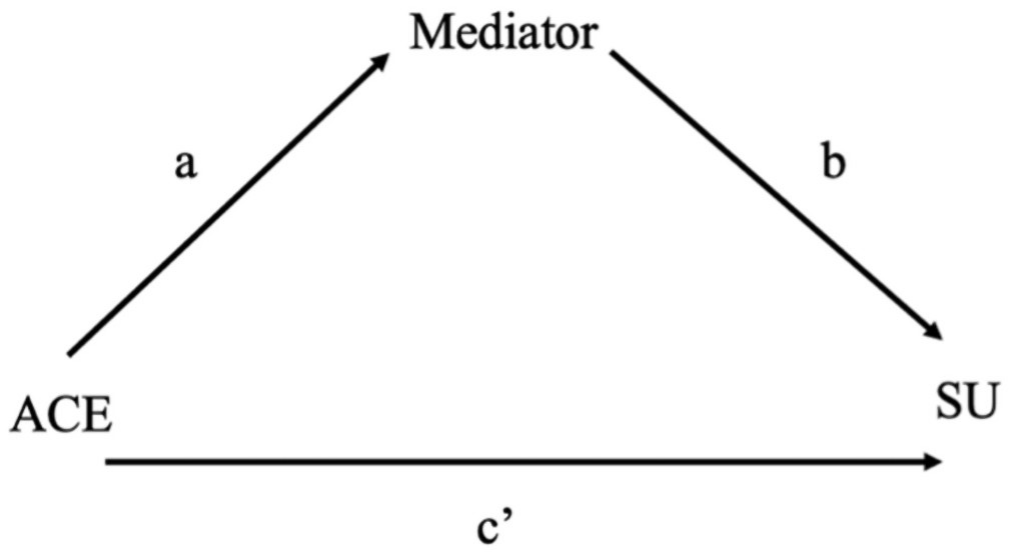
Mediation paths. Representation of direct and indirect effects examined in mediation analysis. The effect of the predictor (ACE) on the mediator corresponds to path a; the effect of the mediator on the substance use outcome corresponds to path b. The indirect effect is the effect of the ACE on the substance use outcome via the mediator, and is the product of paths a and b (ab). The direct effect corresponds to c’ and represents the effect of the ACE on the substance use outcome that does not occur via the mediator. The total effect of the ACE on the substance use outcome is the sum of the indirect (ab) and direct (c’) effects.

As shown in Fig 1, mediation analyses produce both direct and indirect effects from the total effect of a predictor (here, ACE exposure) on an outcome (substance use).

Where available, the standardised indirect effect (ß; the standardised product ab presented in Fig 1) for mediators is reported for ACEs and substance use outcomes that were measured on a continuous scale. This reflects the change in standard deviations in the substance use outcome for each standard deviation change in the ACE (typically severity or frequency). Where the ACE was a dichotomous variable, the partially standardised coefficient is reported, and this is indicated in the results. This is the standard deviation change in substance use between ACE vs. no ACE. Where standardised coefficients were not available, the unstandardised coefficient is reported. The percent of the total effect of the ACE on the substance use outcome that is mediated is presented where available. This was calculated by dividing the indirect effect by the total effect (the sum of the absolute value of indirect and direct effects [21]) and multiplying the result by 100. It can be interpreted as the proportion of the effect of the ACE on the substance use outcome that can be attributed to the mediator.

For moderators, hazard or odds ratios at different levels of the moderator are presented where possible. Where not available, the regression coefficient of the interaction effect was extracted.

### Quality of evidence

Risk of bias was assessed using the Joanna Briggs Institute Critical Appraisal Checklist for Studies Reporting Prevalence Data [22]. The GRADE approach was used to assess the strength of the cumulative evidence [23].

## Results

**Fig 2:**
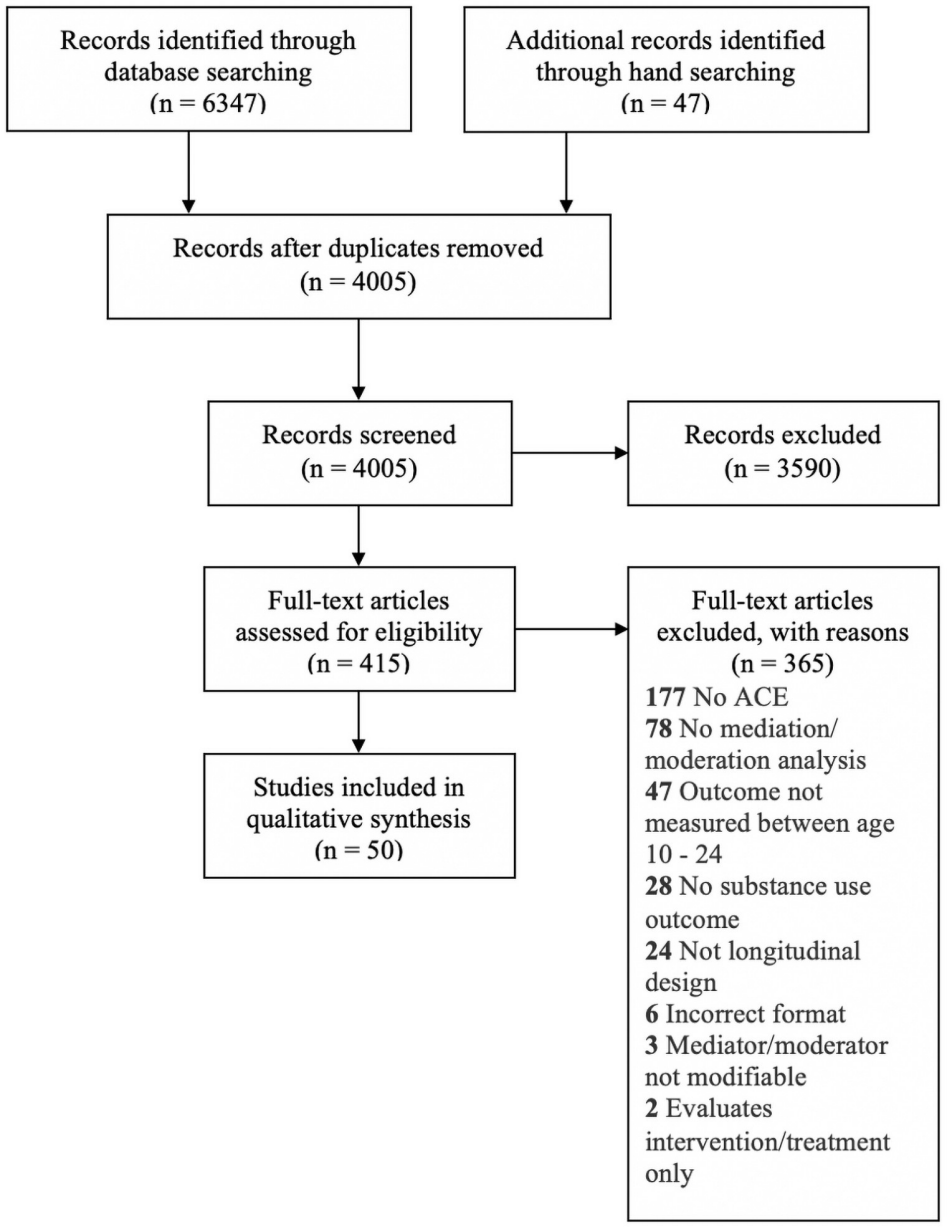
Prisma 2009 flow diagram. Study screening flow chart for studies identified in the systematic review. Titles and abstracts were screened for 4005 studies, resulting in 415 studies for full-text review. Of these, 50 studies were included in the current qualitative synthesis.

Fig 2 presents the study inclusion process. Of the 415 full-text articles reviewed, studies were predominately excluded because they did not meet our criteria for ACEs or did not include a mediation/moderation analysis. No factors were identified at the public policy level of behaviour.

### Mediation analyses

#### 1. Individual-level

*1*.*1 Internalising*. Table 1 presents studies that examined internalising factors. All ACEs studied were positively associated with internalising symptoms [24–27]. However, the impact of internalising symptoms on substance use was mixed. Two studies found a positive association between internalising symptoms and tobacco and substance use respectively [24,27], whereas another found that internalising symptoms were associated with decreased alcohol and cannabis use respectively [25]. In addition, ego over-control, an internalising-type personality trait associated with a tendency to constrain or inhibit emotional impulses, was negatively associated with alcohol abuse symptoms [28]. Two studies found internalising was positively associated with coping motives, which in turn were positively associated with alcohol use, suggesting internalising may increase alcohol use for adolescents who turn to substances to cope. Finally, four studies failed to find evidence for an indirect effect through internalising symptoms [29–32].

**Table 1:** Results of 17 primary studies examining internalising mediators, including pain, depressive symptoms, suicidal ideation, anger, post-traumatic stress symptoms (PTSS), drinking motives, and overall internalising symptoms (a combination of negative affect, anxiety symptoms and somatic complaints).

Mediator	First author and date	n exposed to adversity	Age of ACE exposure (M, years)	Age mediator assessed (M, years)	Age at outcome (M, years)	ACE category	Substance type	Substance outcome	Effect size ß or b (95% CI where available)	Percent mediated	Findings
Pain	Austin 2018 [33]	PA: 3971; SA: 630; PN: 1537	Prior to 6^th^ grade	15	16–22	CM	NMPO	Use	ß = 0.019 (SE = 0.006)	7.57	CM increased probability of pain, which was associated with increased probability of NMPO.
Depressive symptoms	Earnshaw 2017 [34]	NR. Total sample 4297	11	13	16	PV	Alcohol, tobacco, cannabis	Use	b = 0.01 [SE = 0.01; (alcohol, marijuana]; b = 0.02 [SE = 0.01; tobacco]	Unable to calculate (direct effects NR)	PV was associated with increased depressive symptoms, which was associated with increased likelihood of substance use.
Depressive symptoms	Fishbein 2011 [35]	179	<10–12	11–13	14–16	EA	Psycho-active drugs	Initiation by age 16	Probit scale: 0.06 (SE = 0.03)	11.5	Depressive symptoms partially mediated the relationship between EA and onset of drug use. All paths were positive.
Depressive symptoms	Zapolski 2018 [36]	118	12–13	14	15	PV	AOD, tobacco	Use	b = 0.012 (0.001–0.022)	66.18	For females only: peer victimisation predicted depression which predicted substance use. All relationships were positive.
Depressive symptoms	Zoloto 2012 [37]	NR. Total sample = 764.	Median = 13, range = 11–16	Median = 13	~15	Maternal depression	Tobacco	Initiation	b = 0.022 (0.001–0.046)	35.90	Mother’s depression was positively associated with son’s depression, which predicted smoking initiation.
Suicidal ideation	Marschall-Lévesque 2017 [38]	NR. Total sample = 238	13	14	15	PV	Alcohol	Use	b = 0.25 (0.001–0.059)	Unable to calculate (direct effects NR)	Victimisation increased the odds of suicidal ideation, which in turn, predicted higher alcohol use.
Anger	Benedini 2018 [27]	≥365	<12	12	16	SA	Alcohol, tobacco, cannabis	Use	ß = 0.029	48.72	Anger mediated the relationship between SA and substance use. All paths were positive.
Anger	Faulkner 2014 [39]	CM: 128; witness IPV: 61	16	17	18	CM, witness IPV	Alcohol	Problem	CM: b = 0.001 (.0001–0.0046) IPV: b = 0.017 (0.0013–0.0469)	CM = 51.9 IPV = 30.80	Indirect effects from each CM and IPV to problem drinking via anger were significant. All paths were positive direction.
Anger	Kobulsky 2018 [40]	907	<12	16	18	CM	Alcohol, tobacco, cannabis, other psycho-active drugs, inhalants	SU diversity, frequency, presence of disorder	ß: Abuse, boys: 0.06 (0.01, 0.1). Neglect, boys: 0.1 (0.05, 0.15). Neglect, girls: 0.05 (0, 0.1)	Neglect, boys = 28.57, girls = 22.73. Abuse, boys = 52.83	Anger mediated the relationship between neglect and SU severity for both boys and girls, and between child abuse and SU severity for boys only. Both neglect and abuse were associated with increased anger symptoms, which in turn were positively associated with substance use severity.
PTSS	Yoon 2017 [32]	NR. Total sample = 883	<12	12	16	PA, SA	Alcohol, tobacco, cannabis	Use	ß = SA: 0.05 (0.03, 0.08); PA: 0.03 (0.01, 0.05)	SA = 35.71 PA = 33.33	Greater physical and sexual abuse scores were associated with greater PTSS, which in turn were positively associated with adolescent substance use.
PTSS & drinking to regulate emotions	Hannan 2017 [41]	151	<13	19 (PTSS), 20 (drinking motives)	20	SA	Alcohol	Problem drinking	ß (partially standardised = 0.033	Unable to calculate (direct effects NR)	SA was associated with PTSS, which predicted drinking to regulate emotions, which was positively associated with problem drinking. Indirect effect is the combined effect of both mediators.
PTSS + Coping motives	Park 2019 [42]	568	<16	16 (PTSS) 16.5 (coping)	16.5	CM	Alcohol	Problems	ß = 0.03 (0.00, 0.07)	50.00	A serial mediation model was evident, whereby greater frequency of CM predicted increased PTSS, which was associated with increased coping motives, which in turn was associated with greater alcohol problems.
Coping motives	Topper 2011 [43]	NR. Total sample = 324.	13–14	14	15	PV	Alcohol	Problems	b = 0.001 (.0001–.003) ß = 0.024	23.01	Coping motives partially mediated the relationship between PV and alcohol problems. All paths were positive.
Ego over-control	Oshri 2013 [28]	242	<10	10–12	15–18	CM	Alcohol	Abuse/ dependence	ß = -.062 (-0.279, -0.011)	Unable to calculate (direct effects NR)	CM was positively associated with ego overcontrol compared to resilience, which was negatively related to alcohol symptoms. Ego overcontrol did not significantly mediate the path from CM to cannabis abuse.
Internalising + coping expectancy from alcohol	Jester 2015 [26]	468	<8	12–14	18–20	Witness IPV	Alcohol	Use	ß = 0.013	6.1	Witnessing IPV predicted increased alcohol use through INT + coping expectancy. All paths were positive. Indirect effect is the combined effect of both mediators.
Internalising + coping motives	Meisel 2018 [25]	NR. Total N = 387	14	18	19	PV	Alcohol	Use	ß = (0.04, 0.21)	Unable to calculate (direct effects NR)	Chronic peer exclusion was positively associated with INT. INT in turn were negatively associated with alcohol use. INT were positively associated with coping motives, which were positively associated with alcohol use. The indirect effect through INT was significant, as was the indirect effect through INT + coping motives.
Internalising	Meisel 2018 [25]	NR. Total N = 387	14	18	19	PV	Alcohol	Use	ß = (-.43, -.04)	Unable to calculate (direct effects NR)	
Internalising	Benedini 2018 [27]	≥365	<12	14	16	PA, SA	Alcohol, tobacco, cannabis	Use	PA: ß = 0.016 (full sample); 0.045 (girls) SA: 0.017 (full sample), 0.034 (girls)	PA: 12.74 (full sample), 36.00 (girls). SA: 36.17 (full sample), 77.27 (girls)	INT mediated the relationship between PA and SU for both the full sample and just for girls. All paths were positive. INT was not a significant mediator for boys only.
Internalising	Lewis 2011 [24]	422	<12	14	16	CM	Tobacco	Use	ß = 0.06 (0.02, 0.12)	8.96	INT mediated the relationship between CM and smoking. All paths were positive.
**Non-significant results**											
Depressive symptoms	Austin 2018 [33]	PA: 3971; SA: 630; PN: 1537	Prior to 6^th^ grade	15	16–22	CM	NMPO	Use	ß = 0.007	CM predicted greater adolescent depressive symptoms, but depressive symptoms were not significantly associated with NMPO use, and thus did not mediate the path from CM to NMPO use.	
Depressive self-concept (self-esteem)	Bailey 2005 [44]	43	2–14	15	16	SA	AOD	Problem drinking + diversity & freq. of drug use	NR	SA predicted greater depressive self -concept, however depressive self-concept was not significantly associated with substance use.	
Internalising	Handley 2017	163	<11	11	20	CM	Alcohol	Use, problem use	ß = -0.001	Maltreatment did not significantly predict child internalising symptoms or tension reduction alcohol expectancies, nor did internalising symptoms predict alcohol use in emerging adulthood.	
Tension reduction alcohol expectancies	Handley 2017								ß = -0.007		
Internalising	Kobulsky 2016	302	<13	13	15	PA	AOD	Use	ß = −0.007, (−0.031, 0.013)	PA was positively associated with internalising, but internalising was not significantly associated with substance use at follow up.	
Internalising	Proctor 2017 [31]	784	0–6	8	19	CM	Alcohol, cannabis	Initiation	b = 0.0267 (alcohol); b = 0 (cannabis)	Paths from CM to internalising symptoms and from internalising symptoms to substance use initiation were both non-significant.	
Depression	Tartter 2014 [45]	315	<15	15	16–20	Maternal depression	AOD	Disorder	ß = -0.004 (alcohol), ß = 0.17 (cannabis)	Maternal depression was significantly positively associated with adolescent depression; however adolescent depression did not significantly predict later AOD disorder.	
Internalising	Yoon 2017	NR. Total sample = 883	0–12	14	16	CM	Alcohol, tobacco, cannabis	Use	ß = 0	There were no significant indirect effects of the different types of CM on substance use via internalising symptoms.	

Results of original studies identified in the systematic review examining internalising-type mediators of the relationship between ACE exposure and substance use. The top panel presents significant mediators (at p<.05) of the relationship between ACE exposure and substance use; the bottom panel presents factors that were tested but not found to be significant mediators.

Legend: ß: Standardised coefficient; M: Mean; PA: physical abuse; SA: Sexual abuse; PN: Physical neglect; CM: Child maltreatment, includes abuse and neglect; NMPO: Non-medical prescription opioids; AOD: Alcohol and other drugs; BUC: Behavioural under-control; NR: Not reported; PV: Peer victimisation; X: Unable to calculate; IPV: Intimate partner violence; EA: Emotional abuse; F: Frequency; PTSS: Post-traumatic stress symptoms; ER: Emotion regulation; AUD: Alcohol use disorder; INT: Internalising; EXT: Externalising behaviour; CUD: Cannabis use disorder; SU: Substance use; PSU: Parental substance use.

As shown in Table 1, depressive symptoms were identified as mediators by four studies [34–37], while another two studies did not find significant indirect effects through depressive symptoms [33,45]. In all studies, childhood adversity increased depressive symptoms, which increased substance use or increased the likelihood of initiation by mid-late adolescence for four studies [34–37], and was not significantly related to substance use for two studies [33,45]. The percent mediated ranged from 12% to 66%. In addition, suicidal ideation was positively associated with peer victimisation and alcohol use [38].

Approximately one third of the effect of physical and sexual abuse on substance use was mediated by post-traumatic stress symptoms (PTSS) [32]. Two studies found a combined mediated effect of PTSS and drinking motives, whereby PTSS increased drinking to cope or drinking to regulate emotion, in turn predicting increased substance use [41,42]. Drinking to cope was identified by three additional studies as mediating the link between childhood adversity to alcohol use and problem use, and was associated with greater substance use [25,26,43].

Anger was found to mediate the effect of childhood adversity on substance use [27,40] and problem drinking [39]. All instances found ACEs to be positively associated with anger, which was positively associated with substance use outcomes. The percent mediated through anger ranged from 23% to 78%.

1.2 *Externalising*. Table 2 presents studies examining externalising behaviours as mediators. ACE exposure was positively associated with externalising behaviours, which were in turn associated with increased levels of substance use [30,45,46] and a younger age of substance use initiation [31]. The percent mediated through externalising ranged from 14% to 79%.

**Table 2:** Results of ten primary studies examining externalising behaviours as mediators.

Mediator	First author and date	n exposed to adversity	Age of ACE exposure (M, years)	Age mediator assessed (M, years)	Age at outcome (M, years)	ACE category	Substance type	Substance outcome	Effect size ß or b (95% CI where available)	Percent mediated	**Findings**
Externalising	Kobulsky 2016 [30]	302	<13	13	15	PA	AOD	Use	ß = 0.038 (0.017, 0.062)	79.17	PA was positively associated with EXT, which was positively associated with substance use.
Externalising	Proctor 2017 [31]	784	0–6	8	19	CM	Alcohol, cannabis	Initiation	Alcohol: b = -0.158 (−0.29, −0.06); cannabis: b = -0.078 (−0.18 to −0.01)	Alcohol: 40.75; cannabis: 37.5	CM was positively associated with EXT, and EXT was negatively associated with age at initiation of both substances. CM (vs. none) reduced the age of initiation by 0.066 and 0.032 standard deviations for alcohol and cannabis use respectively.
Externalising	Proctor 2017 [31]	164	0–6	8	19	SA	Alcohol, cannabis	Initiation	Alcohol: b = -0.115 (-0.29, -.01); cannabis: b = -0.165 (-0.32, -0.03)	Unable to calculate (direct effects NR)	SA was positively associated with EXT, and EXT was negatively associated with age at initiation of both substances. SA (vs. none) reduced the age of initiation by 0.069 and 0.048 standard deviations for alcohol and cannabis use respectively.
Externalising	Proctor 2017 [31]	562	0–6	8	19	Neglect	Alcohol, cannabis	Initiation	Alcohol: b = -0.098 (−0.20, −0.01); cannabis: b = -0.073 (−0.19 to −0.01)	Unable to calculate (direct effects NR)	Neglect was positively associated with EXT, and EXT was negatively associated with age at initiation of both substances. Neglect (vs. none) reduced the age of initiation by 0.041 and 0.03 standard deviations for alcohol and cannabis use respectively.
Externalising	Tartter 2014 [45]	315	<15	15	16–20	Maternal depression	AOD	Disorder	ß = 0.46 (AUD), 0.43 (CUD)	AUD: 58.12; CUD: 74.14	EXT fully mediated the relationship between maternal depression and diagnosis of AUD and CUD. All paths were positive.
Externalising	Oshri 2011 [46]	259	<7	10–12	13–15	CM	Cannabis	Abuse	ß = 0.021; b = 0.114 (0.014, 0.400)	14.00	CM was positively associated with EXT. CM was also positively associated with ego under-control, which was positively associated with EXT, which was positively associated with cannabis abuse symptoms. Both the indirect effects from CM to cannabis abuse symptoms through EXT, and ego control + EXT, were significant. CM was negatively associated with ego resiliency, which was negatively associated with EXT.
Externalising + Ego resiliency	Oshri 2011 [46]	259	<7	7–12	13–15	CM	Cannabis	Abuse	ß = 0.011; b = 0.062 (0.014, 0.177)	8.16	
Externalising + ego under-control	Oshri 2011 [46]	259	<7	7–12	13–15	CM	Cannabis	Abuse	ß = 0.019; b = 0.103 (0.022, 0.290)	12.84	
Ego under-control	Oshri 2013 [28]	242	<10	10–12	15–18	CM	Cannabis	Abuse/ dependence	ß = 0.055 (0.011–0.329)	37.41	CM was positively associated with ego under-control compared to resilience, which was positively associated with cannabis symptoms. Ego under-control did not significantly mediate the path from CM to alcohol abuse.
Behavioural under-control	Bailey 2005 [44]	43	2–14	15	16	SA	AOD	Problem drinking + diversity & freq. of drug use	ß = 0.150	45.46	SA was positively associated with BUC, which was positively associated with substance use.
Conduct problems	Handley 2017 [29]	163	<11	11	20	CM	Alcohol	Use, problem use	ß = 0.017 (0.005, 0.115)	25.37	Conduct problems mediated relationship between CM and alcohol use. All paths were positive.
Antisocial behaviour	Dishion 1999 [47]	NR. Total sample = 193.	< 9	9	16	PSU	Alcohol	Initiation	X	X	PSU predicted alcohol onset in adolescents. Adolescent antisocial behaviour fully mediated this relationship.
Cognitive Impulsivity	Walters 2018 [48]	395	< 9	13	13.5	PA	AOD	Use	ß = 0.0243, b = 0.237 (0.041–0.592)	79.53	PA predicted increased impulsivity, which predicted greater substance use.
**Non-significant results**											
Externalising	Benedini 2020	≥365	<12	14	16	PA, SA	Alcohol, tobacco, cannabis	Use	NR	Neither PA nor SA were associated with externalising behaviours.	
Externalising	Yoon 2017	NR. Total sample = 883	0–12	14	16	CM	Alcohol, tobacco, cannabis	Use	ß ranges from 0–0.02	There were no significant indirect effects of the different types of CM on substance use via internalising symptoms.	
ADHD symptoms	Zoloto 2012 [37]	NR. Total sample = 764.	Median = 13, range = 11–16	Median = 13	~15	Maternal depression	Tobacco	Initiation	b = -0.03	Adolescent ADHD symptoms did not significantly mediate the path from maternal depression to adolescent smoking.	

Results of original studies identified in the systematic review examining externalising-type mediators of the relationship between ACE exposure and substance use. The top panel presents significant mediators (at p<.05) of the relationship between ACE exposure and substance use; the bottom panel presents factors that were tested but not found to be significant mediators.

ß: Standardised coefficient; M: Mean; PA: Physical abuse; SA: Sexual abuse; CM: Child maltreatment, includes abuse and neglect; AOD: Alcohol and other drugs; BUC: Behavioural under-control; NR: Not reported; X: EA: Emotional abuse; freq.: Frequency; AUD: Alcohol use disorder; INT: Internalising; EXT: Externalising behaviour; CUD: Cannabis use disorder; SU: Substance use; PSU: Parental substance use.

Behavioural under-control, impulsivity, and conduct problems mediated the relationship between childhood adversity and substance use [29,44,48]. All associations were positive, and fully standardised indirect effects ranged from 25% to 80%. Antisocial behaviour fully mediated the relationship between parental substance use and alcohol initiation in adolescence [47].

Externalising behaviour was not found to be a significant mediator of the relationship between maltreatment and substance use in two studies [27,32]. Another study failed to find a significant mediating effect for ADHD symptoms [37].

#### 2. Interpersonal mediators

Table 3 presents studies examining mediators at the interpersonal level. Four studies identified peer factors as mediating the association between childhood adversity and substance use [49–52]. One study found socio-emotional difficulties to mediate the association between parental separation and alcohol use among adolescent girls, but not boys [52]. Three studies included some aspect of peer substance use or delinquency, which significantly mediated the relationship between adversity and was positively associated with substance use [49–51]. Peer deviancy accounted for between 32–63% of the effect of ACEs on substance use [49,50]. However, two studies failed to find a significant mediating effect of affiliation with deviant peers [53,54]. Peer victimisation was not associated with affiliation with deviant peers, and the indirect effect between maternal heavy alcohol use with problem drinking at age 18 years via deviant peers was not significant [53,54].

**Table 3:** Results of 14 primary studies examining interpersonal mediators, including peer and parenting factors.

Mediator	First author and date	n exposed to adversity	Age of ACE exposure (M, years)	Age mediator assessed (M, years)	Age at outcome (M, years)	ACE category	Substance type	Substance outcome	Effect size ß (95% CI where available)	Percent mediated	Findings
Socio-emotional difficulties	Pasqualini 2018	6904	1–8	11	14	Parental separation	Alcohol	Use	X	X	For girls, the indirect effect of parental separation by age 8 on adolescent drinking was mediated by socio-emotional difficulties. For boys, this was not significant.
Peer drug use	Hoffmann 1998 [49]	395	<13	~14	~15	PSUD	AOD	Use	ß = 0.063	31.5	PSUD was positively associated with peer drug use, which was positively related to adolescent AOD use.
Deviant peer affiliation	Yoon 2020 [50]	811	<12	14	16	Emotional abuse	Alcohol, tobacco, cannabis	Use	ß = 0.1	62.5	There was an indirect effect of emotional abuse on substance use via deviant peer affiliation. All associations were positive.
Parental attachment + peer cannabis use/ approval	Mason 2017 [51]	118	2–6: PA, EA, neglect, 2–18: SA	18	18	CM	Cannabis	Use	ß = 0.012 (pre-school CM), 0.016 (SA)	SA: 10.96; pre-school CM: unable to calculate, direct effect NR	CM was negatively associated with parental attachment in adolescence, which in turn was negatively associated with peer cannabis use/approval, which was strongly positively associated with adolescent cannabis use.
Maternal social support	Handley 2013 [55]	~99	12	13	~17	Maternal AUD	Alcohol	Age of initiation	b = -0.095 (-0.17, -0.04)	Unable to calculate, direct effect NR	Mother social support mediated the relationship between current maternal AUD and adolescents’ initiation of alcohol use in the following 4 years. Having a mother with a current AUD diagnosis was associated with receiving less social support in adolescence, which in turn increased the odds of initiating alcohol.
Mother-child relationship	Yoon 2017 [32]	NR. Total sample = 883	0–12	14	16	CM	Alcohol, tobacco, cannabis	Use	ß: EA = 0.024 (0.01, 0.04); Neglect = 0.025 (0.01, 0.05)	EA = 44.44 Neglect = 45.46	Both emotional abuse and neglect were associated with worse mother-child relationship, which in turn was associated with greater substance use.
Positive parenting	Hill 2018 [56]	99	NR. Parent age 21–34*	12	16	Parental CUD	Cannabis	Use	b = 0.319	19.89	Parental CUD was associated with lower positive parenting, which in turn was associated with increased cannabis use.
Positive parenting	Sternberg 2018 [57]	124	<12	12	20	Parental AUD	Alcohol	Use, dependence symptoms	ß = 0.019 (use), 0.040 (dependence)	13.67	Parental AUD was associated with less positive parenting, which in turn predicted early adulthood alcohol consumption. Positive parenting fully mediated this relationship. Higher positive parenting was protective against alcohol consumption.
**Non-significant results**											
Parental attachment	Benedini 2020	≥365	<12	14	16	PA, SA	Alcohol, tobacco, cannabis	Use	NR	Neither PA nor SA were associated with parental attachment.	
Family attachment	Hoffman 1998	395	<13	~14	~15	PSUD	AOD	Use	NR	Parental SUD and affective disorder were significantly negatively associated with family attachment; however family attachment was not related to adolescent AOD at follow up.	
Affiliation with substance-using peers	Dermody 2016 [53]	~290	17	17.5	18	PV	Alcohol	Problem drinking (HED)	ß = -0.008	Victimisation was associated with increased HED, however, victimisation was not associated with affiliation with substance using peers, and therefore substance using peers did not mediate the relationship between victimisation and HED.	
Parental monitoring and affiliation with deviant peers	Mahedy 2018 [54]	553	12	Parental monitoring = 14; deviant peers = 15.5	18	Maternal heavy alcohol use	Alcohol	Problem drinking	Monitoring: b = 0.06 (−0.02, 0.13). Monitoring + peer deviance: b = 0.04 (−0.01, 0.09)	The indirect effect of high-risk maternal alcohol use on young adult problem drinking via parental monitoring was not significant, nor was the indirect effect through parental monitoring and peer deviance.	
Maternal alcohol-specific parenting	Handley 2013 [55]	~99	12	13	~17	Maternal AUD	Alcohol	Age of initiation	NR	Tested maternal alcohol-specific parenting (alcohol disclosure, alcohol legitimacy, strategies to prevent adolescent drinking) and consistency of discipline as mediators. None were significant.	
Cannabis-specific parenting	Sternberg 2019 [58]	99	~5	11.6	16.3	Parental CUD	Cannabis	Use	ß = -0.054 (sharing of neg. experience); ß = -0.041 (parenting to prevent cannabis use).	Parental CUD did not significantly predict cannabis-specific parenting (sharing of negative experiences with cannabis use/ efforts to prevent child from using cannabis). Greater sharing of negative experiences with cannabis were associated with greater odds of an adolescent being a more frequent cannabis user. Parenting strategies to prevent cannabis use were not significantly associate with adolescent cannabis use.	

Results of original studies identified in the systematic review examining interpersonal mediators of the relationship between ACE exposure and substance use. The top panel presents significant mediators (at p<.05) of the relationship between ACE exposure and substance use; the bottom panel presents factors that were tested but not found to be significant mediators.

ß: Standardised coefficient; M: Mean; CM: Childhood maltreatment, includes abuse and/or neglect; EA: Emotional abuse; SA: Sexual abuse; PA: Physical abuse; CUD: Cannabis use disorder; PSUD: Parental substance use disorder; X: Unable to calculate; AUD: Alcohol use disorder; NR: Not reported; AOD: Alcohol and other drugs; PV: Peer victimisation; HED: Heavy episodic drinking.

Five studies found a significant mediating role for parenting factors [32,51,55–57]. Adversity was negatively associated with parental attachment, maternal support, and positive parenting (a composite of monitoring, support and consistency). All mediators demonstrated a protective role of parental support and monitoring against substance use outcomes following exposure to adversity. Between 14% and 45% of the total effect of ACEs on substance use was mediated through parenting quality. However, one study did not find parental monitoring to be a significant mediator of the link from maternal heavy alcohol use to young adult problem drinking [54]. Two other studies found that cannabis-specific and alcohol-specific parenting (sharing of negative experiences and efforts to prevent adolescent use) did not mediate the relationship between parental alcohol or cannabis use disorder and adolescent alcohol or cannabis use [55,58]. Finally, two studies failed to find significant mediation for parental attachment [27,49].

#### 3. Multiple mediators across individual, interpersonal and community levels

Table 4 shows the results of multiple mediation analyses. Two studies found that parental psychopathology influenced later substance use, first via parenting factors including reduced maternal warmth and involved parenting [15,59], which had flow on effects for poorer early childhood self-regulation, late childhood externalising behaviour [15], adolescent affiliation with delinquent or substance-using peers, and favourable youth attitudes to substance use [59]. A further study found cascading effects of school/educational factors, whereby childhood maltreatment was positively associated with school mobility (the number of times a child changed schools) and negatively associated with mid-adolescent reading achievement, which in turn was positively associated with educational attainment by age 22. Educational attainment was protective against tobacco smoking, mediating approximately 28% of the effect of ACEs on smoking [60]. No standardised indirect effects were available.

**Table 4:** Results of three primary studies examining multiple mediators across individual, interpersonal and community levels of behaviour.

Mediator	First author and date	n exposed to adversity	Age of ACE exposure (M, years)	Age mediator assessed (M, years)	Age at outcome (M, years)	ACE category	Substance type	Substance outcome	Percent mediated	Findings
Maternal warmth + self-regulation + EXT + substance- using / delinquent peers	Eiden 2016 [15]	125	1	2 (maternal warmth), 3 (self-regulation), EXT (9–12), peers (13–14)	15–19	Parental AUD	Alcohol, cannabis	Use	Unable to calculate	A significant indirect path was found from parental AUD to adolescent alcohol use via less maternal warmth, poorer self-regulation, greater EXT, increased delinquency/ substance use in early adolescence and increased alcohol use later in adolescence.
Parenting style, substance- using peers, youth attitudes to SU	Murry 2013 [59]	NR. Total sample = 411.	12–13	12–13 (parenting), 17–18 (peers, youth attitudes to SU)	20–21	Maternal psycho-pathology	Alcohol, tobacco, cannabis	Use	Unable to calculate	Maternal psychopathology was negatively associated with involved parenting, which was negatively associated with affiliation with substance using peers, which was positively associated with early-adult substance use. Maternal psychopathology was positively associated with harsh parenting, which was positively associated with substance using peers. Harsh parenting was positively associated with youth favourable attitudes to SU, which was positively associated with early adult substance use.
School mobility, delinquency, reading achievement, socio-emotional skills, educational attainment, adult arrest, life satisfaction	Topitzes 2010 [60]	135	<18	11–22	22–24	CM	Tobacco	Use	School mobility: 14.3; delinquency: 31.4; socio-emotional skills: 14.3; reading achievement: 15.2; educational attainment: 27.6; life satisfaction: 20; adult arrest: 34.3	CM was negatively associated with reading achievement and socio-emotional skills, but positively associated with school mobility, juvenile delinquency and adult arrest. Socio-emotional skills, reading achievement, life satisfaction, and educational attainment were protective against cigarette smoking, whereas juvenile delinquency, school mobility and adult arrest were positively associated with smoking.

Results of original studies identified in the systematic review examining multiple mediators of the relationship between ACE exposure and substance use.

M: Mean; CM: Childhood maltreatment, includes abuse and/or neglect; SU: Substance use; AUD: Alcohol use disorder; EXT: Externalising behaviour.

### Moderation analyses

Ten studies examined modifiable moderators of the relationship between ACEs and later substance use, shown in Table 5. Results of two primary studies that conducted moderated mediation analyses are presented in Supporting Information. As with the mediation analyses, only moderators amenable to psychosocial intervention subsequent to experiencing adversity were eligible for inclusion.

**Table 5:** Results of 10 primary studies that conducted moderation analyses.

Moderator	First author and date	n exposed to adversity	Age ACE exposure (M, years)	Age moderator assessed (M, years)	Age at outcome (M, years)	ACE category	Substance type	Substance outcome	Interaction effect (95% CI where available)	Findings
Religiosity	Chu 2012 [61]	NR. Total sample = 1569.	<14	18	18	SA	Cannabis	Use	X	SA victims were more likely to use marijuana than non-victims, yet the greater the victims’ religiosity the less the likelihood of marijuana use.
Future orientation	Cui 2020 [62]	672	<14	14	18	CM	Alcohol, tobacco, cannabis	Use	ß = -0.158 (−0.245, −0.071)	Youth low in future orientation experienced increased substance use when exposed to CM, compared to youth high in future orientation.
Depression	Woerner 2020 [63]	1059	10–17	10–17	10–17	PV	Alcohol	Initiation	HR: -1SD_dep_: 1.48 (1.17–1.88) +1SD_dep_: 1.12 (0.97–1.30)	The association between PV and alcohol use was stronger at low levels of depression.
Parental mediation of technology use	Wright 2019 [64]	NR. Total sample 867	14	15	17	PV	Alcohol, psycho-active drugs (exc. Cannabis)	Use	ß = -0.19 (alcohol), -0.16 (drugs).	The relationship between PV and substance use was positive. The association between PV and alcohol and other drug use was stronger when parental mediation of technology was low, and was not significant at average levels of parental mediation.
Father-child relationship quality	Dubowitz 2019 [65]	NR. Total sample 702	<12	12–18	12–18	CM	Cannabis	Use	At higher relationship quality, AoR = 0.7 (0.50–0.98)	For teens who had experienced CM, a better-quality relationship with a father was associated with less marijuana use.
Family cohesion, peer drug use, SLEs, self-esteem	Hoffmann 2002 [66]	416	<13	13–20	20	Parental psycho-pathology	AOD	Abuse	X	DA: Higher family cohesion attenuated the effect of parental psychopathology on adolescent DA. PAD is associated with higher risk for DA when SLEs are more common. AA: PSUD and AA were more strongly associated at low levels of peer drug use. PAD was more strongly associated with AA when SLEs were frequent. Higher family cohesion attenuated the relationship between PAD and AA. The relationship between PSUD/ PAD and AA is stronger when self-esteem was high.
Social capital	Kotch 2010 [67]	861	<12	12–16	18	Parental depression	Alcohol, tobacco	Use	b = 0.036 (0.004, 0.068)	When caregiver depression was high, neglected children perceiving a high degree of social cohesion and trust in their neighbourhood showed less alcohol use than those perceiving low social cohesion and trust. This effect was not significant for non-neglected children.
**Non-significant results**										
Positive / negative self-schemas; self-esteem	Corte 2008 [68]	178	3–13	12–14	15–17	Parental AUD	Alcohol	Age of initiation	NR	Tested 2-way interactions between parental AUD and #pos self-schemas; #neg self-schemas; and self-esteem. No interactions were significant. Parental AUD did not predict age of drinking onset or age of first drunkenness.
School engagement	Fulco 2020 [69]	427	8–13	14–17	14–17	Maternal depression	Alcohol, cannabis	Use, problem use	Boys: b = 0.26 (SE = 0.64) Girls: b = 0.89 (SE = 0.58)	Interaction between maternal depressive symptoms and offspring school engagement was not significant, indicating school engagement did not buffer the association between maternal depression and adolescent substance use.
Social support	Feldman 2004 [70]	90	<15	15	19	CM	AOD	Disorder	NR	Interaction between maltreatment and social support was not significant, indicating social support did not moderate the relationship between CM and SUD

Results of original studies identified in the systematic review examining moderators of the relationship between ACE exposure and substance use. The top panel presents significant moderators (at p<.05) of the relationship between ACE exposure and substance use; the bottom panel presents factors that were tested but not found to be significant moderators.

ß: Standardised coefficient; b: Unstandardised coefficient; AoR: Adjusted odds ratio; NR: Not reported; SLEs: Stressful life events; PSUD: Parental substance use disorder; PAD: Parental affective disorder; HR: Hazard ratio; DA: Drug abuse; AA: Alcohol abuse; CM: Child maltreatment (includes abuse and neglect); PV: Peer victimisation; SA: Sexual abuse; dep: Depression; AUD: Alcohol use disorder; #: Number; pos: Positive; neg: Negative; SE: Standard error; SUD: Substance use disorder.

Analyses revealed religiosity and future orientation were each protective of early adult substance use [61,62]. Severity of depression moderated the relationship between peer victimisation and alcohol use initiation, such that this relationship was stronger at low levels of depression [63]. The number of positive and negative self-schemas in adolescence was not found to significantly moderate the association between parental alcohol use disorder and the age of alcohol initiation for offspring [68].

Positive family factors appeared to mitigate the effect of ACE exposure on substance use. Both family cohesion and a stronger relationship between father and child was protective against later substance use and abuse [65,66]. Another study revealed that at low levels of parental regulation of technology use, the relationship between peer victimisation and substance use was stronger, demonstrating a protective effect of this parenting strategy [64]. However, social support (including from family) was not found to be a significant moderator of the relationship between childhood maltreatment and substance use disorder in young adults [70]. In addition, school engagement was not found to be a significant moderator of the relationship between maternal depression and substance use [69].

#### Risk of bias

Risk of bias within studies, assessed using the Joanna Briggs Institute Critical Appraisal Checklist for Studies Reporting Prevalence Data [22], is presented in Supporting Information, along with an assessment of the quality of evidence overall, using the GRADE Approach [23]. The average score for risk of bias within studies was 7 (out of nine, with higher scores indicating a better study, i.e. lower risk of bias). In general, the main risks of bias were non-reporting of the participant sampling methods, and not examining differences between those lost to follow up and those retained in the study.

The overall quality of evidence was moderate. The factors with the strongest evidence of a mediating effect were anger, PTSS, coping motives, externalising, mother-child relationship, and socio-emotional skills. For moderators, the highest rating of quality of evidence was for parental monitoring. There is good evidence that these factors mediate and moderate the relationship between ACEs and substance use by early adulthood.

## Discussion

The current review demonstrated that a substantial proportion of the association between exposure to ACEs and substance use is attributable to subsequent, mediating factors. Multiple mediators and moderators were identified at the individual, interpersonal and community levels of behaviour. This represents the first comprehensive synthesis of factors that mediate and moderate the relationship between ACEs and substance use outcomes by young adulthood (age 24). By extending the research that has established a relationship between exposure and outcome, this study identifies critical intervention targets for the prevention of substance use problems among young people who have experienced childhood adversity.

### Individual factors

Individual mediators fell broadly into internalising and externalising domains, consistently demonstrating that ACE exposure was positively predictive of both, a finding that is supported in the literature [71,72]. Externalising behaviour was associated with worse substance use outcomes, with large mediated effects. Results align with evidence demonstrating externalising behaviours as robust risk factors for the development of substance use problems [73,74], and highlight the importance of addressing externalising behaviour for ACE-exposed youth. Encouragingly, programs that have been shown to effectively reduce externalising behaviours are available. Examples include interventions targeting Attachment, Self-Regulation, and Competency (ARC), which are often affected following exposure to trauma and manifest as behavioural and emotional difficulties [75]. Preliminary evidence shows reductions in externalising scores on the Child Behaviour Checklist [76–78]. Further, a personality-targeted substance use prevention program, Preventure, has demonstrated sustained reductions in externalising behaviours up to two years post-intervention [79].

In contrast, the role of internalising symptoms for substance use was mixed, with some studies [25,28] showing a negative association with substance use, others finding a positive association [24,27], and others finding no evidence of an association [29–32]. Moreover, the four studies that found depression to be a significant mediator found it was positively associated with substance use [34–37]. This inconsistency is similarly discussed in the literature and may reflect differing contributions of the depressive- and anxiety-type symptoms that are encompassed by internalising symptoms [80,81]. This literature shows a more consistent positive relationship between depressive symptoms and substance use, compared to anxiety symptoms and the combined measurement of internalising symptoms [80,82,83]. One plausible interpretation is the role of a tendency to use substances to cope with negative affect [84], one aspect of internalising symptoms. Indeed, five studies from the review found a positive association between endorsing coping motives for drinking and substance use [25,26,41–43]. It may be that adolescents with depressive symptoms may be more likely to seek out substances to cope, whereas those displaying anxiety traits such as social phobic traits are more likely to avoid social contexts that involve substances [85]. Although plausible that young people with anxious traits may be less likely to drink underage [86,87] for fear of engaging in a deviant behaviour and thus, internalising might have a positive relationship with substance use once at older ages, results of the current review show the opposite relationship [24,25,27,28]. Finally, given the comorbidity of internalising and externalising symptoms in adolescence [88], measuring internalising symptoms without controlling for externalising symptoms may erroneously attribute the effects of unmeasured externalising behaviour to internalising symptoms [89].

Results further highlight the importance of addressing anger, PTSS, and suicidal ideation. Anger was associated with increased substance use and problematic use, demonstrating some of the largest mediated effects [27,39,40]. Importantly, anger is associated with poorer substance use treatment outcomes [90], as well as comorbid mental health problems in those with substance use disorders [91]. Taken together, these findings suggest anger is an important individual factor to target among young people exposed to adversity to prevent substance misuse. In addition, results of the current review [32,41,42] align with the “self-medication” hypothesis of co-occurring posttraumatic stress disorder (PTSD) and substance use and emphasise the importance of treating PTSS for improvements in substance use [92]. Screening for PTSS in youth exposed to ACEs is critical, as addressing these early could prevent substance misuse and comorbid PTSD and substance use disorders [93]. Finally, though only one study in the current review examined suicidal ideation as a mediator between ACEs and substance use, suicidal ideation demonstrated a large indirect effect and should of course be screened in this vulnerable population.

### Interpersonal factors

The current review found that ACEs predicted increased substance use through deviant peer affiliation [49–51]. The literature has long identified involvement with deviant peers as being associated with increased substance misuse [73], thus, it is important to understand the function deviant peers are serving in order to address this in prevention. Evidence suggests protection from future victimisation and the need for support and belonging motivates young people to join gangs [94–96], which seems plausible for youth who have experienced abuse or neglect [97]. Research has also identified a role for temperament, in that the presence of more externalising symptoms, such as impulsivity, lower frustration tolerance and self-regulation was predictive of deviant group involvement [98]. Importantly, each of these motives could be addressed in interventions targeted to adolescents. Future research should examine the effectiveness of targeting these motivations to prevent substance misuse.

The current review further highlights the important role of parenting, demonstrating that lower parent-child relationship quality predicted increased substance use, whereas higher family cohesion, relationship quality and parental monitoring attenuated the effect of adversity on substance use outcomes [64–66]. These findings add to the literature on the importance of parenting in substance use prevention [73,74] and suggest prevention programs may benefit from incorporating parent modules focusing on parental support, monitoring, and consistency of discipline [32,55–57]. Of course, improving the parent-child relationship may be contraindicated when a parent is the source of abuse or adversity for the child, however, evidence suggests improved relationship quality with another family member can be protective against problem drinking and smoking [99]. Importantly, for ACE-exposed youth, many of the mediating and moderating factors may be inextricably linked to the adversity (e.g. parental psychopathology and less nurturing relationships), therefore, promoting parental mental health through treatment would likely prevent negative outcomes for the child and prevent ACE exposure altogether [100,101]. In addition, parenting support such as Circle of Security in the post-natal period or Triple P in early childhood could promote parent-child attachment and address externalising behaviours in children [102,103].

### Community factors

This review also demonstrated a role of community factors in the relationship between ACEs and substance use. Impaired educational opportunity and achievement associated with ACE exposure had flow on effects for adult justice involvement and lower educational attainment, which were predictive of increased substance use [60]. Additionally, greater neighbourhood social cohesion and trust was protective against alcohol use among youth exposed to ACEs [67].

**Fig 3:**
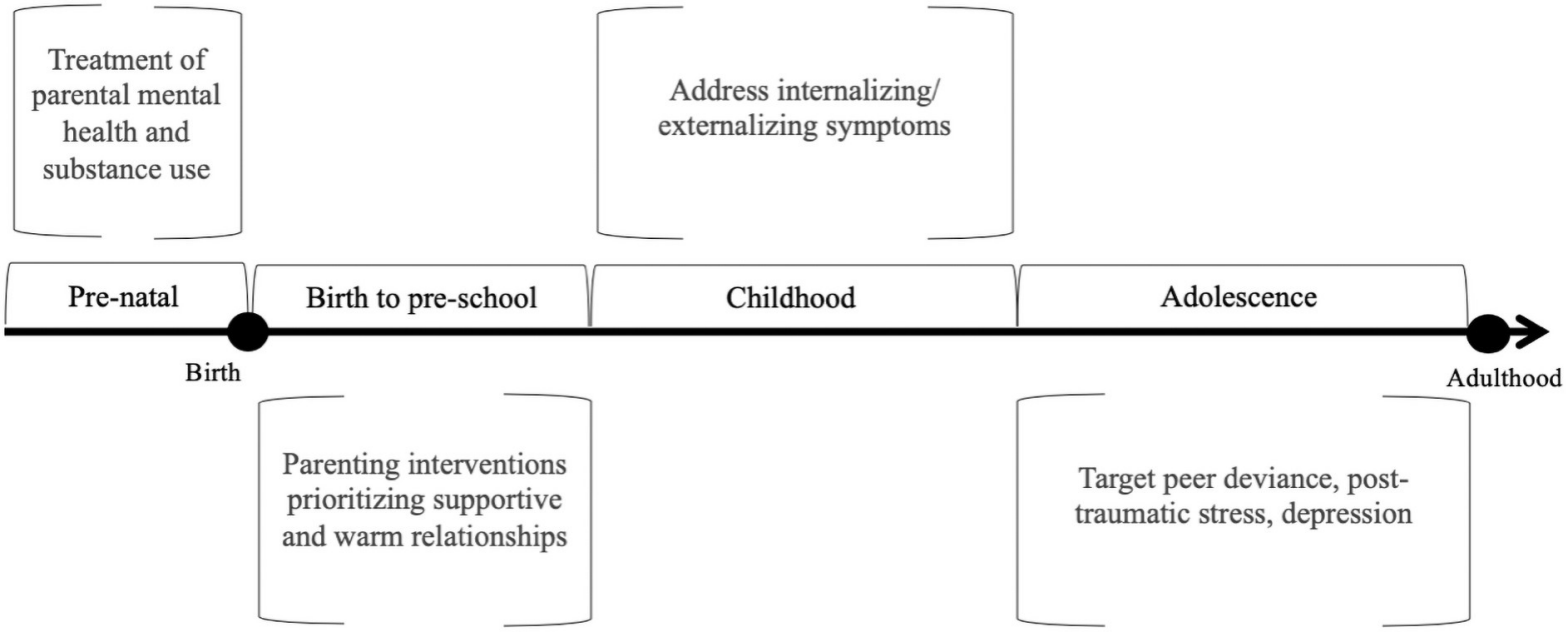
Timeline depicting different opportunities and targets for intervention. Possible timings and targets for intervention to prevent substance misuse in young people exposed to childhood adversity, based on synthesis of the existing literature.

The results highlight multiple opportunities for intervention to prevent substance misuse among young people exposed to ACEs, that can be delivered in either school or healthcare settings. As shown in Fig 3, starting during pregnancy, better treatment of parental substance use and mental health through counselling interventions could prevent ACE exposure altogether [101]. From birth to age 2, interventions to promote supportive and warm relationships with a caregiver should be prioritised, such as Circle of Support parenting and Triple P [102,103]. From childhood, existing school-based programs such as the Preventure program show promise for reducing substance use by targeting internalising and externalising problems and addressing coping skills [104,105]. In addition, implementing strategies to remedy early learning difficulties could prevent the flow on effects of reduced educational attainment, delinquency and substance use problems demonstrated in the current review [60]. The Healthy Environments and Response to Trauma in Schools (HEARTS) program demonstrated improvements in addressing trauma in students, an increase in school engagement and attendance, a reduction in disciplinary incidents including physical aggression, and improvement in trauma-related symptoms for a subsample of students for whom therapy was warranted [106]. By adolescence, peer factors including motivations for deviant affiliation could be targeted. Additionally, schools can refer to external early intervention or treatment services, for example for those suffering from PTSS or suicidal ideation. Importantly, such a model provides numerous targets and stages for intervention, increasing the opportunity to promote healthy development among children exposed to adversity. Given variability in factors such as a child’s response to adversity, the timing of exposure, and their contact with intervention services, such a model is critical to maximise the potential of prevention of harms following ACE exposure.

### Limitations

This study has several limitations. First, given that the review had broad inclusion criteria, there was substantial heterogeneity across studies with respect to their measurement of adversity and outcomes. While this allowed for a broad range of mediators and moderators to be identified, it limited the ability to quantitatively synthesise results. As such, we are only able to present a widely varying range of standardised indirect effects and percent mediated. Second, while the percent mediated statistic is readily interpretable, its use in small sample sizes has been questioned and may be inflated when the total effect is small [107,108]. For some studies included in the review these limitations apply; however, to promote comparability this effect size was reported. These statistics also highlight a need for more research on interaction effects. The high percent mediated for multiple mediators indicate there are unmeasured interactions between variables that work together to explain the link between ACEs and substance use. Although outside the scope of the current review, future research should probe these interactions. Third, no primary studies in the current review examined ACEs such as emotional neglect. Given high prevalence and particular deleterious effects of emotional neglect [109,110], this is an important area for future research. Fourth, the majority of studies were conducted in North America and thus it is unclear the extent to which these findings are generalisable to other contexts, particularly low-middle income countries. Cross-cultural differences in the prevalence of childhood adversity and substance use, attitudes and policy surrounding substance use prevention and treatment, and disparities in resilience have been noted and may limit the relevance of these findings for other cultures [111–115]. Finally, by restricting the searches to only studies published in English, the current review may have missed relevant literature, however, the search terms were re-run without the English language filter and no additional studies were found for inclusion.

### Conclusions and future directions

This review elucidates a range of targets to intervene on the trajectory from ACEs to substance use by early adulthood, including depressive symptoms, anger, PTSS, coping motives, externalising, peer deviance and substance use, and parent relationships. The targets identified in the current review should be used to inform the development of substance use prevention interventions for ACE-exposed youth, or adaptations of existing prevention programs. Indeed, future research should examine whether existing prevention programs that target these factors are effective for youth with histories of adverse experiences, or whether these youth need additional support in these areas.

In addition, future research should address gaps arising from the current review, such as examining mediators linking understudied adversity exposures (e.g., physical and emotional neglect) and substance use. The mechanisms linking different types of adversity exposures (i.e., threat versus deprivation) to psychopathology may be distinct and require tailored interventions [116]. Overall, the review highlights that exposure to adversity in childhood is not a life sentence. Numerous mediators and moderators of the relationship between adversity and substance use point to the complexity of this relationship and offer hope for various intervention points.

## Supporting information

S1 Table PRISMA checklist. (DOCX)

S2 Table Database search terms. (DOCX)

S3 Table Results of two primary studies that conducted moderated mediation analyses. (DOCX)

S4 Table Risk of bias within students, using the Joanna Briggs Institute Critical Appraisal Checklist for Studies Reporting Prevalence Data. (DOCX)

S5 Table The GRADE (Grading of Recommendations, Assessment, Development and Evaluations) approach to assess the strength of the cumulative evidence. (DOCX)
