# Supplementary material for: Targets for intervention to prevent substance use in young people exposed to childhood adversity: A systematic review
Source: PLoS One. 2021 Jun 7;16(6):e0252815. doi: 10.1371/journal.pone.0252815 (PMC8183991; doi:10.1371/journal.pone.0252815)
Supplement: S2 Table — (DOCX) [file pone.0252815.s002.docx]

S2 Table: Database search terms.

| Database: Medline | | |
| --- | --- | --- |
| Childhood Adversity | 1 | Life Change Events/ |
|  | 2 | adverse childhood experiences/ or domestic violence/ or exp child abuse/ or physical abuse/ |
|  | 3 | ((childhood or adolescent) adj3 advers*).tw. |
|  | 4 | (child* or life or early) adj2 stress.tw. |
|  | 5 | bullying/ or cyberbullying/ |
|  | 6 | Social Isolation/ |
|  | 7 | ((Family or parent*) adj3 (substance or alcohol* or drug or smok* or depression or illness or suicid* or jail or prison)).tw. |
|  | 8 | divorce/ or family conflict/ or family separation/ |
|  | 9 | (trauma* or maltreat* or assault* or violen* or molest* or neglect* or victim* or isolat* or reject* or mistreat* or poverty or depriv* or abus* or lonel*).tw. |
|  | 10 | 1 or 2 or 3 or 4 or 5 or 6 or 7 or 8 or 9 |
| Mediator/ moderator | 11 | Resilience, Psychological/ |
|  | 12 | adaptation, psychological/ |
|  | 13 | (adapt* or protect* or resilien* or mediat* or moderat*).tw. |
|  | 14 | protective factors/ |
|  | 15 | 11 or 12 or 13 or 14 |
| Substance use outcomes | 16 | substance-related disorders/ or exp alcohol-related disorders/ or alcoholic intoxication/ or alcoholism/ or binge drinking/ or amphetamine-related disorders/ or cocaine-related disorders/ or drug overdose/ or inhalant abuse/ or marijuana abuse/ or exp opioid-related disorders/ or phencyclidine abuse/ or substance abuse, intravenous/ or substance abuse, oral/ or "tobacco use disorder"/ |
|  | 17 | ((substance or alcohol* or tobacco or drug or smok*) adj3 (misuse* or initiat* or abus* or problem or heavy or binge or disorder* or dependen* or frequen*)).ti,ab. |
|  | 18 | 16 or 17 |
| Study design | 19 | cohort studies/ or longitudinal studies/ or follow-up studies/ or prospective studies/ or retrospective studies/ or cohort.ti,ab. or longitudinal.ti,ab. or prospective.ti,ab. or retrospective.ti,ab. |
|  | 20 | 10 and 15 and 18 and 19 |
| Age range | 21 | Child* or adolescen* or teen* or youth* or pediatr* or paediatr* or young or emerging or youth).tw |
|  | 22 | 20 and 21 |
|  | 23 | limit 22 to (("all child (0 to 18 years)" or "young adult (19 to 24 years)") and english) |
|  | 24 | limit 23 to yr="1998 -Current" |

| Database: PsycINFO | | |
| --- | --- | --- |
| Childhood Adversity | 1 | exp childhood adversity/ |
|  | 2 | ((childhood or adolescent) adj3 advers*).tw. |
|  | 3 | ((child* or life or early) adj2 stress).tw. |
|  | 4 | bullying/ or cyberbullying/ |
|  | 5 | social isolation/ |
|  | 6 | ((Family or parent*) adj3 (substance or alcohol* or drug or smok* or depression or illness or suicid* or jail or prison)).tw. |
|  | 7 | divorce/ or marital separation/ or life changes/ |
|  | 8 | exp family conflict/ |
|  | 9 | (trauma* or maltreat* or assault* or violen* or molest* or neglect* or victim* or isolat* or reject* or mistreat* or poverty or depriv* or abus* or lonel*).tw. |
|  | 10 | child abuse/ or abandonment/ or child neglect/ or child welfare/ or domestic violence/ or emotional abuse/ or physical abuse/ or sexual abuse/ |
|  | 11 | 1 or 2 or 3 or 4 or 5 or 6 or 7 or 8 or 9 or 10 |
| Mediator/ moderator | 12 | "resilience (psychological)"/ |
|  | 13 | exp social adjustment/ |
|  | 14 | (adapt* or protect* or resilien* or mediat* or moderat*).tw. |
|  | 15 | protective factors/ |
|  | 16 | adaptive behaviour/ |
|  | 17 | 12 or 13 or 14 or 15 or 16 |
| Substance use outcomes | 18 | exp drug abuse/ or addiction/ or drug addiction/ or drug overdoses/ or intravenous drug usage/ or prescription drug misuse/ |
|  | 19 | exp "substance use disorder"/ |
|  | 20 | exp Alcoholism/ or exp Alcohol Abuse/ or exp Drug Dependency/ |
|  | 21 | binge drinking/ or alcohol intoxication/ |
|  | 22 | ((substance or alcohol* or tobacco or drug or smok*) adj3 (misuse* or initiat* or abus* or problem or heavy or binge or disorder* or dependen* or frequen*)).ti,ab. |
|  | 23 | exp marijuana usage/ or "cannabis use disorder"/ |
|  | 24 | cocaine/ |
|  | 25 | exp amphetamine/ |
|  | 26 | exp opiates/ or "opiod use disorder"/ |
|  | 27 | exp "Tobacco Use Disorder"/ |
|  | 28 | 18 or 19 or 20 or 21 or 22 or 23 or 24 or 25 or 26 or 27 |
| Study design | 29 | cohort studies/ or longitudinal studies/ or follow-up studies/ or prospective studies/ or retrospective studies/ or cohort.ti,ab. or longitudinal.ti,ab. or prospective.ti,ab. or retrospective.ti,ab. |
| Age range | 30 | (Child* or adolescen* or teen* or youth* or pediatr* or paediatr* or young or emerging or youth).tw. |
|  | 31 | 11 and 17 and 28 and 29 and 30 |
|  | 32 | limit 31 to (english and human) |

| Database: PubMed | | |
| --- | --- | --- |
| Child adversity | 1 | ((((((((((((life change events[MeSH Terms]) OR adverse childhood experiences[MeSH Terms]) OR domestic violence[MeSH Terms]) OR physical abuse[MeSH Terms]) OR bullying[MeSH Terms]) OR social isolation[MeSH Terms]) OR divorce[MeSH Terms]) OR family conflict[MeSH Terms]) OR family separation[MeSH Terms]) OR child* advers*[Text Word]) OR adolescen* advers*[Text Word]) OR ((family[Text Word] OR parent*)[Text Word] AND (substance[Text Word] OR alcohol*[Text Word] OR drug[Text Word] OR smok*[Text Word] OR depressi*[Text Word] OR illness[Text Word] OR suicid*[Text Word] OR jail[Text Word] OR prison)[Text Word])) OR (trauma*[Text Word] OR maltreat*[Text Word] OR assault*[Text Word] OR violen*[Text Word] OR molest*[Text Word] OR neglect*[Text Word] OR victim*[Text Word] OR isolat*[Text Word] OR reject*[Text Word] OR mistreat*[Text Word] OR poverty[Text Word] OR depriv*[Text Word] OR abus*[Text Word] OR lonel*[Text Word]) |
| Mediators | 2 | (((("Resilience, Psychological"[Mesh]) OR "Adaptation, Psychological"[Mesh]) OR "Protective Factors"[Mesh])) OR ((adapt*[Text Word] OR protect*[Text Word] OR resilien*[Text Word] OR mediat*[Text Word] OR moderat*[Text Word])) |
| Substance use outcomes | 3 | ((((substance[Title/Abstract] OR alcohol*[Title/Abstract] OR tobacco[Title/Abstract] OR drug[Title/Abstract] OR smok*)[Title/Abstract] AND (misuse*[Title/Abstract] OR initiat*[Title/Abstract] OR abus*[Title/Abstract] OR problem[Title/Abstract] OR heavy[Title/Abstract] OR binge[Title/Abstract] OR disorder*[Title/Abstract] OR dependen*[Title/Abstract] OR frequen*))[Title/Abstract])) OR (("Substance-Related Disorders"[Mesh]) OR "Binge Drinking"[Mesh]) |
| Study type | 4 | ((cohort[Title/Abstract] OR longitudinal[Title/Abstract] OR prospective[Title/Abstract] OR retrospective[Title/Abstract])) OR ((((("Cohort Studies"[Mesh]) OR "Follow-Up Studies"[Mesh]) OR "Prospective Studies"[Mesh]) OR "Longitudinal Studies"[Mesh]) OR "Retrospective Studies"[Mesh]) |
| Age range | 5 | (Child*[Text Word] OR adolescen*[Text Word] OR teen*[Text Word] OR youth*[Text Word] OR pediatr*[Text Word] OR paediatr*[Text Word] OR young[Text Word] OR emerging[Text Word] OR youth[Text Word]) |
|  | 6 | 1 and 2 and 3 and 4 and 5 |
|  | 7 | Filters activated: Humans, English, Child: birth-18 years, Young Adult: 19-24 years. |

| Database: Web of Science | | |
| --- | --- | --- |
|  | 9 | (#7 AND #6 AND #5 AND #4 AND #3) AND LANGUAGE: (English) |
|  |  | Indexes=SCI-EXPANDED, SSCI, A&HCI, CPCI-S, CPCI-SSH, ESCI, CCR-EXPANDED, IC Timespan=All years |
|  | 8 | #7 AND #6 AND #5 AND #4 AND #3 |
| Age range | 7 | ALL=( child*  OR  adolescen*  OR  teen*  OR  youth*  OR  pediatr*  OR  paediatr*  OR  young  OR  emerging  OR  youth ) |
| Study type | 6 | ALL=( "cohort studies"  OR  "longitudinal studies"  OR  "follow-up studies"  OR  "prospective studies"  OR  "retrospective studies"  OR  cohort  OR  longitudinal  OR  prospective  OR  retrospective ) |
| Substance use outcomes | 5 | ALL=((("substance-related disorders" OR "alcohol-related disorders" OR "alcoholic intoxication" OR "alcoholism" OR "amphetamine-related disorders" OR "cocaine-related disorders" OR "binge drinking" OR "drug overdose" OR "inhalant abuse" OR "marijuana abuse" OR "opioid-related disorders" OR "substance abuse" OR "tobacco use disorder") OR ((substance OR alcohol* OR tobacco OR drug OR smok*) "NEAR" (misuse* OR initiat* OR abus* OR problem OR heavy OR binge OR disorder* OR dependen* OR frequen*)))) |
| Mediator/moderator | 4 | ALL=(resilien*or adapt* or protect* or mediat* or moderat*) |
|  | 3 | #2 OR #1 |
| Child adversity | 2 | ALL=(((child* OR adolescen*) "NEAR" advers*) OR ((child* OR life OR early) "NEAR" stress) OR ((family OR parent*) "NEAR" (substance OR alcohol* OR drug OR smok* OR depressi* OR illness OR suicid* OR jail OR prison) OR (trauma* OR maltreat* OR assault* OR violen* OR molest* OR neglect* OR victim* OR isolat* OR reject* OR mistreat* OR poverty OR depriv* OR abus* OR lonel*))) |
|  | 1 | ALL=("life change events"  OR  "adverse childhood experiences"  OR  "domestic violence"  OR  "child abuse"  OR  "physical abuse"  OR  "sexual abuse"  OR  bullying  OR  cyberbullying  OR  "social isolation"  OR  divorce  OR  "family conflict"  OR  "family separation" ) |

| Database: CINAHL | | |
| --- | --- | --- |
|  | S23 | S10 AND S14 AND S18 AND S21 AND S22 |
| Age ranges | S22 | Child* or adolescen* or teen* or youth* or pediatr* or paediatr* or young or emerging or youth |
|  | S21 | S19 OR S20 |
| Study type | S20 | cohort or longitudinal or prospective or retrospective |
|  | S19 | (MH "Prospective Studies+") |
|  | S18 | S15 OR S16 OR S17 |
| Substance use outcomes | S17 | ((substance or alcohol* or tobacco or drug or smok*) N3 (misuse* or initiat* or abus* or problem or heavy or binge or disorder* or dependen* or frequen*)) |
|  | S16 | (MH "Overdose") |
|  | S15 | (MH "Substance Use Disorders+") |
|  | S14 | S11 OR S12 OR S13 |
| Mediator/ moderator | S13 | adapt* or protect* or resilien* or mediat* or moderat* |
|  | S12 | (MH "Adaptation, Psychological") |
|  | S11 | (MH "Hardiness") |
|  | S10 | S1 OR S2 OR S3 OR S4 OR S5 OR S6 OR S7 OR S8 OR S9 |
| Child adversity | S9 | (trauma* or maltreat* or assault* or violen* or molest* or neglect* or victim* or isolat* or reject* or mistreat* or poverty or depriv* or abus* or lonel*) |
|  | S8 | ((Family or parent*) N3 (substance or alcohol* or drug or smok* or depression or illness or suicid* or jail or prison)) |
|  | S7 | ((child* or adolescen*) N3 advers*) |
|  | S6 | ((child* or life or early) N2 stress) |
|  | S5 | (MH "Family Conflict") |
|  | S4 | (MH "Social Isolation+") |
|  | S3 | (MH "Bullying+") OR (MH "Cyberbullying") |
|  | S2 | (MH "Life Change Events") OR (MH "Death+") OR (MH "Divorce") |
|  | S1 | (MH "Adverse Childhood Experiences") OR (MH "Domestic Violence+") OR (MH "Exposure to Violence") OR (MH "Child Abuse+") OR (MH "Divorce") OR (MH "Poverty") |
|  |  | Limiters - English Language; Age Groups: Adult: 19-44 years, All Child |
