# Supplementary material for: Targets for intervention to prevent substance use in young people exposed to childhood adversity: A systematic review
Source: PLoS One. 2021 Jun 7;16(6):e0252815. doi: 10.1371/journal.pone.0252815 (PMC8183991; doi:10.1371/journal.pone.0252815)
Supplement: S3 Table — (DOCX) [file pone.0252815.s003.docx]

1. Results of moderated mediation analyses.

Moderated mediation exists when the effect of a mediator is different depending on the level of the moderator. This results in a conditional indirect effect, that is, the indirect effect via the mediator is conditional on levels of the moderator [1]. Two such studies were present in the current review, shown in Table 5. The standardised indirect effect is for the effect of the mediator only, while the conditional indirect effect quantifies the indirect effect at a particular level of the moderator. Analyses revealed that the influence of both internalising symptoms and deviant peer affiliation on substance use differed depending on levels of neighbourhood disorder and parental monitoring respectively [2, 3]. That is, when neighbourhood disorder was low, the relationship between internalising symptoms and substance use was significant, however at high levels of neighbourhood disorder it was not [2]. In addition, at high levels of parental monitoring, girls were less likely to affiliate with deviant peers, and for both sexes, the relationship between associating with deviant peers and alcohol use was weaker than at lower levels of parental monitoring.

S3 Table: Results of two primary studies that conducted moderated mediation analyses.

| **Mediator** | **Moderator** | **First author & date** | **n exposed to ACE** | **Age exposed (years)** | **Age m, w, assessed (M, years)** | **Age at outcome (M, years)** | **ACE category** | **Substance type** | **Outcome** | **ß (95% CI), % mediated** | **Conditional indirect effect** | **Findings** |
| --- | --- | --- | --- | --- | --- | --- | --- | --- | --- | --- | --- | --- |
| INT | Neighbour-hood disorder | Duprey 2017 [2] | 300 | 0-4 | 14 | 16 | Neglect | Alcohol, tobacco, cannabis | Use | 0.017 (0.002, 0.039) | 0.008 (0.00, 0.02): low neighbour-hood disorder | High INT was associated with greater likelihood of substance use at low levels of neighbourhood disorder. |
| Deviant peer affiliation | Parental monitoring | Jiang 2016 [3] | NR. Total sample = 723. | 12-13 | 14 | 14 | PV | Alcohol, tobacco | Use | Alc: 0.023, 60%  Smk: 0.066, 75% (boys only) | X | Deviant peer affiliation fully mediated the effect of PV on alcohol use, and for smoking for boys. For both genders, monitoring was protective against alcohol use: when monitoring was high, the association between deviant peers and alcohol use was weaker than when it was low. For boys with low monitoring, deviant peer affiliation was associated with smoking. |

m: mediator; w: moderator; ß: standardised indirect effect; INT: internalising symptoms; PV: peer victimisation.

References:

1. Preacher KJ, Rucker DD, Hayes AF. Addressing Moderated Mediation Hypotheses: Theory, Methods, and Prescriptions. Multivariate Behavioral Research. 2007 2007/06/29;42(1):185-227.

2. Duprey EB, Oshri A, Caughy MO. Childhood Neglect, Internalizing Symptoms and Adolescent Substance Use: Does the Neighborhood Context Matter? J Youth Adolesc. 2017;46(7):1582-97.

3. Jiang Y, Yu C, Zhang W, Bao Z, Zhu J. Peer victimization and substance use in early adolescence: Influences of deviant peer affiliation and parental knowledge. Journal of Child and Family Studies. 2016;25(7):2130-40.
