# Supplementary material for: Targets for intervention to prevent substance use in young people exposed to childhood adversity: A systematic review
Source: PLoS One. 2021 Jun 7;16(6):e0252815. doi: 10.1371/journal.pone.0252815 (PMC8183991; doi:10.1371/journal.pone.0252815)
Supplement: S4 Table — (DOCX) [file pone.0252815.s004.docx]

S4 Table: Risk of bias within studies, using the Joanna Briggs Institute Critical Appraisal Checklist for Studies Reporting Prevalence Data [1].

| Reviewer One | | | | | | | | | | | | |
| --- | --- | --- | --- | --- | --- | --- | --- | --- | --- | --- | --- | --- |
|  |  | 1 | 2 | 3 | 4 | 5 | 6 | 7 | 8 | 9 | Total | Notes |
| Austin | 2018 | Y | Y | Y | Y | Y | Y | Y | Y | Y | 9 |  |
| Bailey | 2005 | Y | Unclear | N | Y | Y | Y | Y | Y | N | 6 | Item 2 - random sampling not reported. Item 3 - unlikely, number exposed to ACE = 43. Item 9 - Those lost to follow-up were more likely to have been recruited from battered women's shelters and have more family stressors. |
| Benedini | 2020 | Y | Y | Unclear | N | N | Y | Y | Y | Y | 6 | Item 3 - number exposed to maltreatment NR, though at least 365 were exposed to some maltreatment. Item 4 - number exposed NR. Item 5 - more females missing data, less likely exposed to physical abuse, lower parental attachment, more externalising problems. |
| Chu | 2012 | N | Y | Unclear | N | Y | Y | Y | Y | N | 5 | Items 3 & 4 - unclear how many of the sample were exposed to CSA. Item 9 - data analysis conducted with 45% of the baseline sample. |
| Crawford | 2008 | Y | N | N | N | Y | Y | Y | Y | Unclear | 5 | Item 2 - oversampled Asian and Latino. Adjusted for this in Stata. Item 3 - small sample exposed. Item 4 - little description provided around study setting, recruitment, sampling etc. Item 9 - attrition NR. |
| Cui | 2020 | Y | Y | Y | Y | Y | Y | Y | Y | Unclear | 8 | Item 9 - differences between those lost to follow up and those retained are not reported. 33% drop out by age 16 |
| Dishion | 1999 | Y | Unclear | N | N | Y | Y | Y | Y | Y | 6 | Item 2 - random sampling not reported. Item 3 - total sample small. Item 4 - unclear what proportion of the sample experienced parental substance use, unclear of ages of children at predictor variables. Item 4 - Item 6 - predominately yes, however 1 item in the parental substance use measure included using drugs to fall asleep, have more energy, relieve pain etc. Unclear whether this reflects abuse. |
| Dubowitz | 2019 | Y | Y | Y | N | N | Y | Y | Y | Y | 7 | Item 4 - unclear number of participants who experienced CM. Item 5 - 50% of the original sample had data on inclusion criteria. |
| Duprey | 2017 | Y | Unclear | N | Y | Y | Y | Y | Y | Unclear | 6 | Item 2 - sampling not reported. Item 3 - sample size < 500. Item 9 - 38% attrition over 12 years, but differences between lost to follow-up and retained groups NR. |
| Earnshaw | 2017 | Y | Y | Y | Y | Y | Y | Y | Y | Y | 9 |  |
| Eiden | 2016 | Y | Y | N | Y | Y | Y | Y | Y | Y | 8 | Item 3 - small sample exposed. |
| Faulkner | 2014 | Y | Y | N | Y | N | Y | Y | Y | N | 6 | Item 3 - small sample exposed. Item 5 - 62% female sample. Item 9 – those lost to follow-up more likely to be male, non-Crown Ward youth. Did not differ on predictor or outcome variables. 28% attrition. |
| Fergusson | 2008 | Y | Y | N | N | N | Y | Y | Y | Y | 6 | Item 3 - small sample exposed. Item 4 - information on the exact final n included in this analysis, n exposed, and the gender breakdown not provided. Item 5 - slight but significant underrepresentation of those from lower SES backgrounds. This was accounted for statistically by using probability of study participation weights. The impact of missing data was evaluated by re-running the analysis using imputed values, producing the same conclusions as reported. |
| Fishbein | 2011 | Y | Y | N | N | Y | Y | Y | Y | Unclear | 6 | Item 3 - small sample exposed. Item 4 - gender breakdown not provided, mean age at each wave not provided, only age range. Item 9 - authors state data is MAR but do not mention attrition statistics or results of any comparison between those included in analysis and those lost to follow up. |
| Handley | 2013 | Y | Y | N | N | Y | Y | Y | Y | Unclear | 6 | Item 3 - small sample exposed. Item 4 - age at follow up not presented. Item 9 - response rate not reported, authors simply report missing data was handled with full information maximum likelihood, but do not report any comparison of those lost to follow up vs. those included in the analysis. |
| Handley | 2017 | Y | Y | N | N | Y | Y | Y | Y | Unclear | 6 | Item 3 - small sample exposed. Item 4 - country not provided. Item 9 - attrition not reported. |
| Hannan | 2017 | N | N | N | Y | Y | Y | Y | Y | N | 5 | Item 1 - used undergraduate psychology students, which does not cover the whole target population e.g. college students not representative of general population of young adults. Item 2 - convenience sampling. Item 3 - small sample exposed. Item 9 - 56% of baseline sample completed final wave, those lost to follow up were less likely to be White. |
| Hill | 2018 | Y | Unclear | N | Y | N | Y | Y | Y | Y | 6 | Item 2 - random sampling not reported. Item 3 - small sample exposed. Item 5 - attrition was low (16% across 9 years), however those lost to follow-up were older and more likely to have a parent who had used cannabis but did not meet criteria for CUD. |
| Hoffmann | 1998 | Y | Y | N | N | Y | Y | Y | Y | Y | 7 | Item 3 - sample size < 500. Item 4 - not clear as to what age children were at each follow-up. |
| Hoffmann | 2002 | Y | Unclear | Y | Y | Y | Y | Y | Y | Y | 8 | Item 2 - sampling not reported. |
| Jester | 2015 | Y | Unclear | Y | Y | N | Y | Y | Y | Unclear | 6 | Item 2 - sampling not reported. Item 5- only 31% of sample were female. Item 9 - attrition not reported. |
| Jiang | 2016 | Y | Unclear | Y | Y | Y | Y | Y | Y | Y | 8 | Item 2 - sampling was not reported, authors simply state 3 middle schools in China. Unknown whether these are representative of Chinese teenagers. |
| Kobulsky | 2016 | Y | Y | N | Y | Y | Y | Y | Y | Unclear | 7 | Item 3 - sample size < 500. Item 9 - attrition not reported. |
| Kobulsky | 2018 | Y | Y | Y | Y | Y | Y | Y | Y | Y | 9 |  |
| Kotch | 2010 | Y | Y | Y | Y | Y | Y | Y | Y | Y | 9 |  |
| Krohn | 2009 | N | Unclear | N | Y | Y | Y | Y | Y | Y | 6 | Item 1 - boys and families in high-crime areas were oversampled, to get a sample of youths at high-risk for delinquency, yet the study generalises to the effects of family transitions on children generally. Item 2 - specifics of sampling not reported. Item 3 - small sample exposed. |
| Lewis | 2011 | Y | Y | Y | Y | Y | Y | Y | Y | Y | 9 |  |
| Marshall-Levesque | 2017 | Y | Y | N | Y | N | Y | Y | Y | Y | 6 | Item 3 - sample size was low, n = 238, and NR the number of children exposed to victimisation (likely small). Item 5 - Approached 1000 families, 572 agreed to participate, only had available data for 238. |
| Mason | 2017 | Y | Unclear | N | Y | Y | Y | Y | Y | Y | 7 | Item 2 - random sampling not reported, some recruitment was done through CPS records, but doesn't say how the rest were sampled and whether they used all CPS records to identify a sample or just some. Item 3 - small sample exposed. |
| Meisel | 2018 | Y | Y | N | Y | Y | Y | Y | Y | Y | 8 | Item 3 - total sample 387, sample exposed NR. |
| Murry | 2013 | Y | Unclear | N | Y | Y | Y | Y | Y | Unclear | 6 | Item 2 - sampling not reported. Item 3 - sample size < 500. Item 9 - attrition not reported. |
| Oshri | 2011 | Y | Y | N | N | Y | Y | Y | Y | Y | 7 | Item 3 - small sample exposed. Item 4 - country of study not reported. |
| Oshri | 2013 | Y | Y | N | N | Y | Y | Y | Y | Y | 7 | Item 3 - small sample exposed. Item 4 - country of study not reported. |
| Park | 2020 | Y | Y | Y | Y | Y | Y | Y | Y | Y | 9 |  |
| Proctor | 2017 | Y | Unclear | Y | Y | Y | Y | Y | Y | Y | 8 | Item 2 - sampling not reported. |
| Sternberg | 2018 | Y | Unclear | N | Y | Y | Y | Y | Y | N | 6 | Item 2 - sampling NR. Item 3 - small sample exposed. Item 9 - around 37% of the sample was excluded. Those excluded were significantly younger, and had higher levels of positive parenting. |
| Tartter | 2014 | Y | Y | N | Y | Y | Y | Y | Y | Unclear | 7 | Item 3 - sample size < 500. Item 9 - differences in those included in analysis and those not included not reported. 816 were selected, but only 702 included in analysis. |
| Topitzes | 2010 | Y | Y | N | Y | N | Y | Y | Y | Y | 7 | Item 3 - small sample exposed. Item 5 – those lost to follow-up had slightly less females and African Americans. 9 - These differences were statistically controlled for through multivariate analyses and propensity scoring procedures entered into secondary regressions testing for the effects of differential attrition. |
| Topper | 2011 | Y | Unclear | N | Y | N | Y | Y | Y | Y | 6 | Item 2 - sampling not reported, had 18 schools across London. Item 3 - sample size < 500. Item 5 - 28% male. |
| Walters | 2018 | Y | Unclear | N | Y | N | N | Y | Y | N | 4 | Item 2 - sampling not reported. Item 3 - sample size < 500. Item 5 - only 37% of the sample had complete data on all variables. Item 9 - did not report differences for those lost to follow up |
| Woerner | 2020 | Y | Y | Y | Y | Y | Y | Y | Y | Y | 9 |  |
| Wright | 2019 | Y | Y | Y | N | Y | Y | Y | Y | Y | 8 | Item 4 - unclear how many participants endorsed being victimised. |
| Yoon | 2017 | Y | Y | Y | Y | Y | Y | Y | Y | Y | 9 |  |
| Yoon | 2020 | Y | Y | Y | Y | Y | Y | Y | Y | Unclear | 8 | Item 9 - differences between those included and excluded in final analysis are not reported. |
| Zapolski | 2018 | Y | Unclear | N | Y | Y | Y | Y | Y | Unclear | 6 | Item 2 - whether sampling was random or not is not reported. Item 3 - small sample exposed. Item 9 - attrition not reported, comparison of groups included vs. excluded not reported. |
| Zoloto | 2012 | Y | Unclear | Y | N | Y | Y | Y | Y | N | 6 | Item 2 - random sampling not reported. Item 9 - those lost to follow-up were more likely to smoke and have parents/friends who smoke |

| Reviewer Two | | | | | | | | | | | | |
| --- | --- | --- | --- | --- | --- | --- | --- | --- | --- | --- | --- | --- |
|  |  | 1 | 2 | 3 | 4 | 5 | 6 | 7 | 8 | 9 | Total | Notes |
| Austin | 2018 | y | y | y | y | y | y | y | y | y | 9 |  |
| Bailey | 2005 | y | NR | n | y | y | y | y | y | y | 7 | 9 - Lower participation from those originally recruited from battered women’s shelters or with more family stressors at baseline. Only girls who participated in all three waves of data collection were included |
| Benedini | 2020 | y | y | n | y | n | y | y | y | y | 7 | 3 - 47% of 777 reported physical abuse, so only 365; 9 - The full information maximum likelihood (FIML) method was used to address missing data |
| Chu | 2012 | y | y | NR | y | y | y | y | y | n | 7 | 3 - 1,569 participants in this study, but did not report the percentage reporting CSA; 9 – 45% lost to follow-up though no significant difference was found between these groups on demographics |
| Crawford | 2008 | y | y | NR | n | y | y | y | y | NR | 6 | 2 - individuals of Asian and Latino descent oversampled. Adjusted using National Center for Education Statistics (NCES) panel weights. 3 - did not report prevalence of ACE; 9 - did not report on response rate, although did account for missing data by imputation |
| Cui | 2020 | y | y | y | y | y | y | y | y | y | 9 | response rates were fairly good, and they determined the data was missing at random ( but they did not discuss reasons for non-response or compare persons in the study to those not in the study) |
| Dishion | 1999 | y | NR | n | y | y | y | y | y | y | 7 |  |
| Dubowitz | 2019 | y | y | y | y | y | y | y | y | y | 9 |  |
| Duprey | 2017 | y | NR | y | y | y | y | y | y | y | 8 | 9 - accounted for missing data - assumed missing at random |
| Earnshaw | 2017 | y | y | y | y | y | y | y | y | y | 9 |  |
| Eiden | 2016 | y | y | n | y | y | y | y | y | y | 8 |  |
| Faulkner | 2014 | y | y | n | y | n | y | y | y | n | 6 | 5 & 9 - More females remained in the study as did Crown ward youth compared to those classified under other CPS status. |
| Fergusson | 2008 | y | y | n | y | y | y | y | y | y | 8 | initial sample size decent size but hard to know if it is sufficient as prevalence of ACEs not reported; response rate okay and accounted for in analyses |
| Fishbein | 2011 | y | y | n | y | y | y | y | y | NR | 7 |  |
| Handley | 2013 | y | y | n | n | y | y | y | y | NR | 6 |  |
| Handley | 2017 | y | y | n | n | y | y | y | y | n | 6 | 9 - only 61% of initial sample completed wave 2 |
| Hannan | 2017 | n | n | n | y | y | y | y | y | n | 5 | 1 - first year psychology students may not be representative of female college students; 9 - response rate low for third wave, attrition was examined but not accounted for |
| Hill | 2018 | y | NR | n | y | n | y | y | y | y | 6 | 5 - more attrition for those with parents with cannabis use |
| Hoffmann | 1998 | y | n | n | y | y | y | y | y | y | 7 | 2 - convenience sample; 9 - very high retention rate |
| Hoffmann | 2002 | y | n | n | y | y | y | y | y | y | 7 | 2 - convenience sample; 3 - 416 from PSUD and AD families; 9 - very high retention rate |
| Jester | 2015 | y | NR | y | y | n | y | y | y | NR | 6 | 5 - low percentage of female participants |
| Jiang | 2016 | y | y | y | y | y | y | y | y | y | 9 |  |
| Kobulsky | 2016 | y | y | n | y | y | y | y | y | NR | 7 |  |
| Kobulsky | 2018 | y | y | y | y | y | y | y | y | n | 8 | 9 - those lost to follow-up more likely to have experienced child abuse than those in the analysis |
| Kotch | 2010 | y | y | y | y | y | y | y | y | y | 9 |  |
| Krohn | 2009 | n | NR | n | y | y | y | y | y | y | 6 | 1 - specific population; 9 - Between Waves 2 and 10, only had 1 percent attrition each year |
| Lewis | 2011 | y | y | y | y | y | y | y | y | y | 9 |  |
| Marshall-Levesque | 2017 | y | y | n | y | y | y | y | y | y | 8 | 9 – majority (60%) had complete data. Missing data was associated with lower parental education and considered missing at random |
| Mason | 2017 | y | NR | n | y | y | y | y | y | y | 7 |  |
| Meisel | 2018 | y | y | n | y | y | y | y | y | y | 8 |  |
| Murry | 2013 | y | NR | n | y | y | y | y | y | y | 7 | 9 – Good retention and no differences between groups on demographics. |
| Oshri | 2011 | y | y | n | n | y | y | y | y | y | 7 | 4- study setting not reported |
| Oshri | 2013 | y | y | n | n | y | y | y | y | y | 7 | 4- study setting not reported |
| Park | 2020 | y | y | y | y | y | y | y | y | y | 9 |  |
| Proctor | 2017 | y | NR | y | y | y | y | y | y | y | 8 |  |
| Sternberg | 2018 | y | NR | n | y | y | y | y | y | NR | 6 |  |
| Tartter | 2014 | y | y | n | y | y | y | y | y | NR | 7 | 3 - 315 mothers had depression |
| Topitzes | 2010 | y | y | n | y | n | y | y | y | y | 7 | 5 – Those included in analysis more likely females, African Americans, and CPC preschool participants. 9 - sample differences controlled for in multivariate analyses and used propensity scoring to predict probability of inclusion and entered this variable into regression models to test effects of differential attrition. |
| Topper | 2011 | y | NR | n | y | n | y | y | y | y | 6 | 5 - 71.7% female. 9 – good follow up rate at 12 months, males more likely to drop out but analyses controlled for gender |
| Walters | 2018 | y | NR | n | y | n | y | y | y | y | 6 | 5 - only included those with complete data on all variables which was around 37%. |
| Woerner | 2020 | n | y | y | y | y | y | y | y | y | 8 | 1 - oversampled low-income neighbourhoods, so may not fit for general population. |
| Wright | 2019 | y | y | NR | y | y | y | y | y | y | 8 | 3 - did not report prevalence of victimisation aggression. 6 - measure seems okay, although more aggression/victimisation than bullying. |
| Yoon | 2017 | y | y | y | y | y | y | y | y | y | 9 |  |
| Yoon | 2020 | y | y | y | y | y | y | y | y | n | 8 | 9 - 60% retention. Differences between those included and excluded in final analysis not reported |
| Zapolski | 2018 | y | NR | n | y | y | n | y | y | NR | 5 | 6 - measure of peer victimisation but not bullying specifically. |
| Zoloto | 2012 | y | NR | y | y | y | y | y | y | n | 7 | 9 - 70% retention at each wave. Those lost to follow up more likely to be smokers and have parents/friends who smoke.. |

NR: not reported

References:

1. Munn Z, Moola, S., Lisy, K., Riitano, D., Tufanaru, C. Methodological guidance for systematic reviews of observational epidemiological studies reporting prevalence and incidence data. Int J Evid Based Healthc. 2015;13(3):147-53.
