# Supplementary material for: Targets for intervention to prevent substance use in young people exposed to childhood adversity: A systematic review
Source: PLoS One. 2021 Jun 7;16(6):e0252815. doi: 10.1371/journal.pone.0252815 (PMC8183991; doi:10.1371/journal.pone.0252815)
Supplement: S5 Table — (DOCX) [file pone.0252815.s005.docx]

S5 Table: The GRADE (Grading of Recommendations, Assessment, Development and Evaluations) approach to assess the strength of the cumulative evidence [1].

| **Outcomes** | **Number of**  **participants** | **Number of**  **primary studies** | **Serious**  **ROB** | **Inconsistency** | **Indirectness** | **Imprecision** | **Range of percent**  **mediated effects** | **Large effect**  **size** | **No plausible**  **confounding** | **Quality of the evidence**  **(GRADE)** |  | **Comment** |
| --- | --- | --- | --- | --- | --- | --- | --- | --- | --- | --- | --- | --- |
| **Mediators** |  |  |  |  |  |  |  |  |  |  |  |  |
| Pain | ~3971 | 1 | No | None - only 1  primary study | None | No | 8 |  |  | Moderate | 3 | Only 1 primary study |
| Depressive  symptoms | ~5358 | 4 | No | No | No | Yes | 12 - 66 | 1 |  | High | 4 | Small samples in two of the studies, with small direct effects, may inflate the percent mediated. Wide range of percent mediated. Graded up for large effect. |
| Suicidal  ideation | ~238 | 1 | Some | None - only 1  primary study | No | No | NA |  | 1 | Moderate | 3 | Only 1 primary study and some risk of bias. Graded up for no temporal confounding. |
| Anger | ~1400 | 3 | No | No | No | Yes | 23 - 78 | 1 |  | High | 4 | Wide range in percent mediated. Graded up for large effect. |
| PTSS | ~1602 | 3 | No | No | No | No | 33 - 50 | 1 |  | High | 4 |  |
| Coping  motives | ~1898 | 5 | No | No | No | No | 6 - 50 | 1 |  | High | 4 |  |
| Internalizing* | ~1884 | 5 | No | Some | No | Yes | 6 - 77 | 1 |  | Moderate | 3 | Inconsistent direction of mediator, wide range in percent mediated. Graded up for large effect. |
| Externalizing** | 2628 | 9 | No | No | No | Yes | 8 - 80 | 1 |  | High | 4 | Wide range in percent mediated. Graded up for large effect. |
| Antisocial  behaviour | ~193 | 1 | Some | None - only 1  primary study | No | Some | NA |  |  | Low | 2 | Only 1 primary study and some risk of bias |
| Substance-using  peers | ~1256 | 6 | No | No | No | Yes | 11 - 70 | 1 |  | High | 4 | Wide range in percent mediated. Graded up for large effect. |
| Peer  deviancy | 956 | 2 | No | Some | No | No | 18 - 63 | 1 | 1 | High | 4 | Some inconsistency - only significant for boys in one of the studies. Graded up for large effect and no temporal confounding. |
| Parental  attachment | 118 | 1 | No | None - only 1  primary study | No | Some | 11 |  |  | Low | 2 | Only 1 primary study and some imprecision given small sample size |
| Mother-child  relationship | ~1107 | 3 | No | No | No | No | 44 - 45 | 1 | 1 | High | 4 |  |
| Positive  parenting | ~634 | 3 | Some | No | No | No | 14 - 20 | 1 |  | High | 4 | Some risk of bias. Graded up for large effect. |
| Favourable  attitudes to SU | ~411 | 1 | Some | None - only 1  primary study | No | No | NA |  |  | Low | 2 | Only 1 primary study and some risk of bias |
| Delinquency | 135 | 1 | No | None - only 1  primary study | No | Some | 31 - 34 | 1 |  | Moderate | 3 | Only 1 primary study with small sample size. Graded up for large effect. |
| Socio-emotional  skills | 260 | 2 | No | No | No | No | 14.3 |  |  | High | 4 |  |
| Education | 135 | 1 | No | None - only 1  primary study | No | Some | 14 - 28 | 1 |  | Moderate | 3 | Only 1 primary study and small sample size. Graded up for large effect. |
| Life  satisfaction | 135 | 1 | No | None - only 1  primary study | No | Some | 20 | 1 |  | Moderate | 3 | Only 1 primary study and small sample size. Graded up for large effect. |
| **Moderators** |  |  |  |  |  |  | **Risk or protective factor** |  |  |  |  |  |
| Religiosity | ~1569 | 1 | Yes | None - only 1  primary study | No | No | Protective |  |  | Low | 2 | Only 1 primary study and risk of bias |
| Future  orientation | 672 | 1 | No | None - only 1  primary study | No | No | Protective |  |  | Moderate | 3 | Only 1 primary study |
| Depression | 1059 | 1 | No | None - only 1  primary study | Some | No | Protective |  |  | Low | 2 | Only 1 primary study, and some indirectness. The relationship between ACE and SU was stronger at low levels of depression, so high levels of depression would seemingly protect against SU due to the ACE. But this is not the purpose of the research question nor practically useful. |
| Parental  monitoring | ~1590 | 2 | No | No | No | No | Protective |  |  | High | 4 |  |
| Father-child  relationship | ~702 | 1 | No | None - only 1  primary study | No | No | Protective |  |  | Moderate | 3 | Only 1 primary study |
| Family  cohesion | 416 | 1 | No | None - only 1  primary study | No | No | Protective |  |  | Moderate | 3 | Only 1 primary study |
| Substance-using  peers | 416 | 1 | No | None - only 1  primary study | Some | No | Protective |  |  | Low | 2 | Only 1 primary study and some indirectness. The relationship between ACE and SU was stronger at low levels of peer substance use, so high levels of peer substance use would seemingly protect against SU due to the ACE. But this is not the purpose of the research question nor practically useful. |
| Stressful life  events | 416 | 1 | No | None - only 1  primary study | No | No | Risk |  |  | Moderate | 3 | Only 1 primary study |
| Self-esteem | 416 | 1 | No | None - only 1  primary study | Some | No | Risk |  |  | Low | 2 | Only 1 primary study and some indirectness with regard to the research question. High levels of self-esteem were associated with a stronger relationship between ACE and SU, yet this may be because at lower levels of self-esteem they are using substances regardless of exposure. Reducing self-esteem to weaken the relationship between ACE and SU is not practically useful. |
| Neighbourhood  social  capital | 1161 | 2 | No | Some | Some | No | Protective |  |  | Low | 2 | Inconsistency between studies and some indirectness. In better neighbourhoods, INT was positively associated with substance use, suggesting that in worse neighbourhoods it is not INT that predicts SU but rather some characteristic of the neighbourhood. |

* Includes internalising symptoms and ego over-control
** Includes externalising, ego under-control, behavioural under-control, conduct problems, and cognitive impulsivity

References:

1. Guyatt GH, Oxman AD, Vist GE, Kunz R, Falck-Ytter Y, Alonso-Coello P, et al. GRADE: an emerging consensus on rating quality of evidence and strength of recommendations. BMJ. 2008;336(7650):924.
